# Sonocatalytic hydrogen/hole-combined therapy for anti-biofilm and infected diabetic wound healing

**DOI:** 10.1093/nsr/nwad063

**Published:** 2023-03-06

**Authors:** Qingqing Xu, Shengqiang Chen, Lingdong Jiang, Chao Xia, Lingting Zeng, Xiaoqing Cai, Zhaokui Jin, Shucun Qin, Wenjiang Ding, Qianjun He

**Affiliations:** Taishan Institute for Hydrogen Biomedical Research, School of Basic Medical Sciences, The Second Affiliated Hospital of Shandong First Medical University & Shandong Academy of Medical Sciences, Tai’an 271000, China; Guangdong Key Laboratory for Biomedical Measurements and Ultrasound Imaging, School of Biomedical Engineering, Medical School, Shenzhen University, Shenzhen 518060, China; Guangdong Key Laboratory for Biomedical Measurements and Ultrasound Imaging, School of Biomedical Engineering, Medical School, Shenzhen University, Shenzhen 518060, China; Guangdong Key Laboratory for Biomedical Measurements and Ultrasound Imaging, School of Biomedical Engineering, Medical School, Shenzhen University, Shenzhen 518060, China; Guangdong Key Laboratory for Biomedical Measurements and Ultrasound Imaging, School of Biomedical Engineering, Medical School, Shenzhen University, Shenzhen 518060, China; Guangdong Key Laboratory for Biomedical Measurements and Ultrasound Imaging, School of Biomedical Engineering, Medical School, Shenzhen University, Shenzhen 518060, China; Shanghai Key Laboratory of Hydrogen Science & Center of Hydrogen Science, School of Materials Science and Engineering, Shanghai Jiao Tong University, Shanghai 200240, China; Guangdong Key Laboratory for Biomedical Measurements and Ultrasound Imaging, School of Biomedical Engineering, Medical School, Shenzhen University, Shenzhen 518060, China; Guangdong Key Laboratory for Biomedical Measurements and Ultrasound Imaging, School of Biomedical Engineering, Medical School, Shenzhen University, Shenzhen 518060, China; Taishan Institute for Hydrogen Biomedical Research, School of Basic Medical Sciences, The Second Affiliated Hospital of Shandong First Medical University & Shandong Academy of Medical Sciences, Tai’an 271000, China; Shanghai Key Laboratory of Hydrogen Science & Center of Hydrogen Science, School of Materials Science and Engineering, Shanghai Jiao Tong University, Shanghai 200240, China; Taishan Institute for Hydrogen Biomedical Research, School of Basic Medical Sciences, The Second Affiliated Hospital of Shandong First Medical University & Shandong Academy of Medical Sciences, Tai’an 271000, China; Guangdong Key Laboratory for Biomedical Measurements and Ultrasound Imaging, School of Biomedical Engineering, Medical School, Shenzhen University, Shenzhen 518060, China; Shanghai Key Laboratory of Hydrogen Science & Center of Hydrogen Science, School of Materials Science and Engineering, Shanghai Jiao Tong University, Shanghai 200240, China

**Keywords:** nanocatalytic medicine, hydrogen therapy, biofilm, diabetic foot, piezoelectric catalysis

## Abstract

It is a great challenge to effectively eradicate biofilm and cure biofilm-infected diseases because dense extracellular polymeric substance matrix prevents routine antibacterial agents from penetrating into biofilm. H_2_ is an emerging energy-regulating molecule possessing both high biosafety and high tissue permeability. In this work, we propose a concept of sonocatalytic hydrogen/hole-combined ‘inside/outside-cooperation’ anti-biofilm for promoting bacteria-infected diabetic wound healing based on two-dimensional piezoelectric nanomaterials. Proof-of-concept experiments using C_3_N_4_ nanosheets as a representative piezoelectric catalyst with wide band gap and high biosafety have verified that sonocatalytically generated H_2_ and holes rapidly penetrate into biofilm to inhibit bacterial energy metabolism and oxidatively deprive polysaccharides/NADH in biofilm to destroy the bacterial membrane/electron transport chain, respectively, inside/outside-cooperatively eradicating biofilm. A bacteria-infected diabetic wound model is used to confirm the excellent *in vivo* antibacterial performance of sonocatalytic hydrogen/hole-combined therapy, remarkably improving bacteria-infected diabetic wound healing. The proposed strategy of sonocatalytic hole/hydrogen-combined ‘inside/outside-cooperation’ will make a highway for treatment of deep-seated biofilm infection.

## Teaser

A concept of sonocatalytic hydrogen/hole-combined ‘inside/outside-cooperation’ anti-biofilm is proposed for efficient hydrogen generation and local polysaccharide/NADH oxidation, which inhibits bacterial aspiration inside biofilm and destroys the surface structure of biofilm, respectively.

## INTRODUCTION

**Scheme 1.**
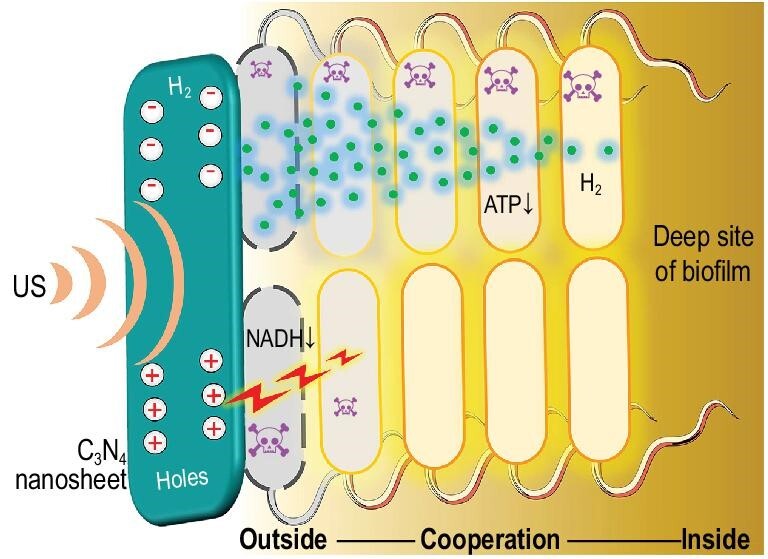
Schematic illustration of the strategy and mechanism of sonocatalytic hydrogen/hole-combined ‘inside/outside-cooperation’ anti-biofilm.

Biofilm is a collective of microbial cells surrounded with dense extracellular polymeric substance (EPS) matrix with high heterogeneity and complexity, which prevents antibacterial agents from penetration into the deep region of biofilm, frequently leading to high drug resistance [1–3]. Most pathogenic microorganisms can form biofilm and make significant contributions to human diseases, but no specific targeting drugs are available so far [4,5]. Recently, photocatalytic oxidation has been developed as an emerging antibacterial method, which mainly destroys the structure of bacterial membrane through oxidation, but hardly affects the internal structure and bacteria inside biofilm, resulting in limited efficacy of anti-bacteria treatment and rare application in anti-biofilm [6–18]. Therefore, we here proposed a strategy of sonocatalytic hydrogen/hole-combined ‘inside/outside-cooperation’ anti-biofilm. As illus-trated in Scheme 1, low-intensity medical ultrasound (US), with a higher tissue penetrability than light, excitated piezoelectric nanomaterials to generate hydrogen molecules (H_2_) and holes, which play a role of fighters inside and outside of biofilm castles, respectively, for cooperative anti-biofilm. H_2_ was used as a Trojan horse to easily penetrate into the biofilm castle for cooperating with hole fighters outside the biofilm castle.

The existence of biofilm at the infected wound will greatly impede wound healing [19,20]. Typically, the high glucose environment of a diabetic foot ulcer is subject to inducing bacterial infection and biofilm formation, which leads to long-term severe ulceration and difficult healing of a diabetic foot wound [21,22]. Anti-biofilm on the diabetic foot wound is one of the important routes to promote diabetic wound healing [22,23], but is still a very challenging problem. Therefore, we here innovatively proposed the catalytic hydrogen/hole production strategy to eradicate biofilm and thus promote infected diabetic wound healing.

In this work, based on the proposed strategy of sonocatalytic hydrogen/hole-combined ‘inside/outside-cooperation’ anti-biofilm, we developed a kind of piezoelectric C_3_N_4_ nanosheets loading hydrogel (C_3_N_4_@Gel) as a US probe-couplable catalyst for sonocatalytic hydrogen production by utilizing polysaccharides/NADH in biofilm as a sacrificial agent. As illustrated in Scheme 1, sonocatalytic polysaccharides/NADH deprivation and hydrogen production destroyed the bacterial membrane/electron transport chain and depressed biofilm energy metabolism, respectively, jointly playing an efficient anti-biofilm effect and consequently promoting the healing of an infected diabetic wound. Noticeably, H_2_ rapidly penetrated into the inside of biofilm for anti-bacteria purposes, which cannot be achieved by routine antibacterial agents including reactive oxygen species (ROS) with a short life time and a short diffusion distance. Compared with light waves for photocatalytic anti-biofilm, US wave has remarkably higher tissue penetrability and lower toxicity to normal cells at low intensity, and sonocatalytic efficiency is much higher and also enables easier catalytic hydrogen generation for combined anti-biofilm with more candidates of catalysts, especially those which have a large band width such as C_3_N_4_ [6–10].

## RESULTS AND DISCUSSION

### Preparation, characterization and sonocatalytic hydrogen production performance of C_3_N_4_ nanosheets

**Figure 1.**
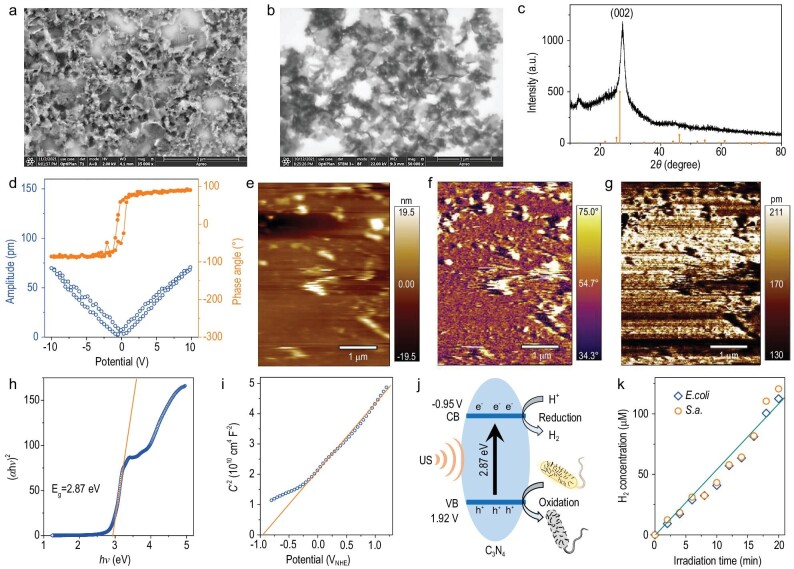
Morphology, structure, piezoelectricity and sonocatalytic hydrogen production performance characterizations of C_3_N_4_ nanosheets. SEM (a) and STEM (b) images of C_3_N_4_ nanosheets, the XRD pattern (c), the hysteresis loops (d), the piezoresponse height (e), phase (f) and amplitude (g) patterns, the curve of (*αhν*)^2^ *vs hv* (h), the Mott−Schottky curve (i), the schematic illustration of band structure (versus NHE (normalized hydrogen electrode)) and sonocatalytic H_2_ production and bacterial oxidation (j), the time-dependent sonocatalytic performances of C_3_N_4_ nanosheets (k).

C_3_N_4_ bulk with a graphitic structure and high piezoelectricity was firstly prepared on a large scale using urea as raw material by using a thermal polycondensation method [8], and then exfoliated into C_3_N_4_ nanosheets by an ultrasonic crushing method in order to obtain higher surface area and flexibility in favor of piezoelectric catalysis [24–28]. As shown in Supplementary Fig. S1, as-synthesized C_3_N_4_ bulk was a kind of micron-sized particle constructed by stacking multilayer sheets. After ultrasonic exfoliation, C_3_N_4_ presented a morphology of thin nanosheets, possessing a higher specific surface area (Fig. 1a and b). Atomic force microscope (AFM) results further confirmed that the thickness of C_3_N_4_ nanosheets was only 10−18 nm (Supplementary Fig. S2). High surface area can provide an abundance of reaction sites for catalysis, while thinner nanosheets morphology has higher flexibility and piezoelectricity with higher performance of piezoelectric catalysis [29]. Furthermore, from elemental mapping results, both C and N elements were uniformly distributed in C_3_N_4_ nanosheets, revealing that ultrasonic exfoliation did not destroy its molecular structure (Supplementary Fig. S3) in consistence with XRD structure characterization results (Fig. 1c, JCPDS#87-1526). The slight shift in the (002) diffraction peak was possibly due to the decrease of C_3_N_4_ layer thickness.

The piezoelectric property of C_3_N_4_ nanosheets was analyzed by piezoelectric force microscopy (PFM). As shown by hysteresis loops in Fig. 1d, C_3_N_4_ exhibited a phase angle change of ∼180° and an amplitude change of ∼75 nm under the inversion of 10 V direct current bias field, indicating that synthesized C_3_N_4_ nanosheets had excellent piezoelectric effect. Moreover, the piezoresponse amplitude and phase patterns also clearly demonstrated visible contrasts (Fig. 1e−g), further confirming the piezoelectricity of C_3_N_4_ nanosheets [30].

Next, the energy band structure of C_3_N_4_ nanosheets was measured to check the feasibility of sonocatalytic hydrogen generation. The absorption spectrum of C_3_N_4_ nanosheets was firstly measured by UV spectroscopy (Supplementary Fig. S4), and then their band gap was calculated to be 2.87 V using the conventional Tauc equation (Fig. 1h). Furthermore, the conduction band (CB) of C_3_N_4_ nanosheets was detected to be −0.95 V with the Mott−Schottky curve (Fig. 1i), and the band structure was illustrated as demonstrated in Fig. 1j. It can be found that catalytically generated electrons and holes held enough high redox potentials to reduce H^+^ into H_2_ and oxidize both bacterial/biofilm polysaccharide (+0.43 V) and bacterial NADH (+0.32 V) in theory [31,32].

The sonocatalytic hydrogen generation behaviors of C_3_N_4_ nanosheets in different bacterial suspensions were examined using a medical ultrasonic physiotherapy instrument (1.0 MHz, 1 W/cm^2^, 50% duty cycle). As shown in Fig. 1k, 2 mg/mL C_3_N_4_ nanosheets sonocatalytically produced more than 110 μM H_2_ after 20 min irradiation of US, meaning that both *Escherichia coli* (*E.coli*) and *Staphylococcus aureus* (*S.a.*) bacteria can be used as sacrificial agents for sonocatalytic hydrogen generation in accordance with the above-mentioned energy band results. Moreover, the amount of H_2_ produced was almost linearly dependent on the US irradiation time, and both types of bacteria at the same concentration exhibited almost the same rate of H_2_ production (Fig. 1k). These results indicated that C_3_N_4_ nanosheets can stably generate H_2_ and simultaneously oxidize bacteria in a sonocatalytic way.

### Antibacterial and anti-biofilm behaviors of sonocatalytic hydrogen-hole combination

**Figure 2.**
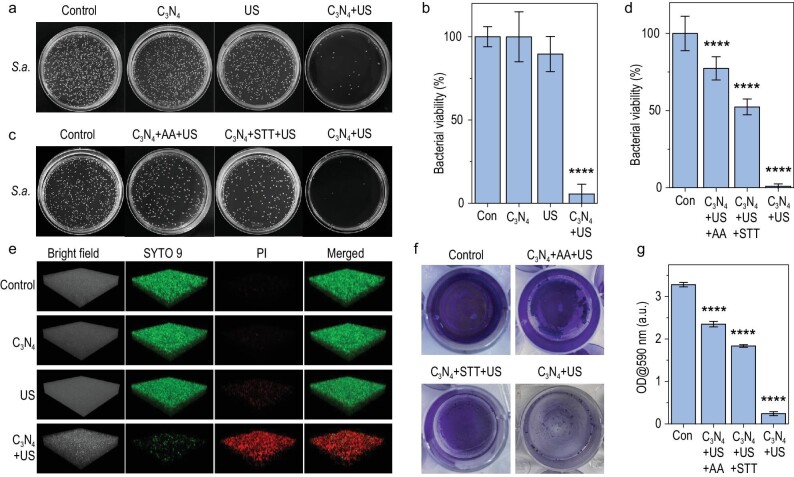
*In vitro* sonocatalytic hydrogen/hole-combined antibacterial and anti-biofilm performances of C_3_N_4_ nanosheets. Digital photographs of *S.a.* bacterial colonies in the agar plate (a and c) and corresponding statistical analyses (*n* = 3 biologically independent samples) (b and d), three-dimensional (3D) confocal images of *S.a.* biofilms (e), digital photographs of crystal violet stained *S.a.* biofilm (f) and corresponding statistical analysis (*n* = 3 biologically independent samples) (g). Con, control. *P* values were calculated by the one-way ANOVA method (****P < 0.0001).

Based on the confirmation of the sonocatalytic hydrogen production and bacterial oxidation performance of C_3_N_4_ nanosheets *in vitro*, the sonocatalytic antibacterial performance of C_3_N_4_ nanosheets was further evaluated with two representative types of bacteria, *E.coli* (Gram-negative) and *S.a.* (Gram-positive). As shown in Fig. 2a and b, and Supplementary Fig. S5, C_3_N_4_ nanosheets almost did not affect the bacterial viability of both *E.coli* and *S.a.*, and US irradiation alone had a weak bacteriostatic effect, which was probably caused by ultrasonic cavitation [33]. In contrast, sonocatalytic therapy with C_3_N_4_ + US (1.0 MHz, 1 W/cm^2^, 50% duty cycle) showed significant antibacterial outcomes against both *E.coli* and *S.a.* (Fig. 2a and b, and Supplementary Fig. S5). With the extension of US irradiation time, the survival rates of *E.coli* and *S.a.* kept decreasing, and both of them were almost completely eradicated after 20 min (Supplementary Fig. S6), suggesting the US time dependence of sonocatalytic therapy outcome. This indicated that sonocatalytic therapy with C_3_N_4_ nanosheets can efficiently deactivate bacteria in spite of bacterial types, and the sonocatalytic therapy outcome can be maximized by adjusting the time duration of US irradiation.

Furthermore, to determine the individual contributions of sonocatalytically generated hydrogen molecules and holes, ascorbic acid (AA) and Na_2_S_4_O_6_ (STT) were used as hole- and electron-sacrificial agents to investigate the antibacterial effect of only hydrogen molecules and holes, respectively [34]. In the concentration range of 0−1000 μM, neither AA nor STT affected the activity of *S.a.* and *E.coli* bacteria (Supplementary Figs S7 and S8), so the concentration of 1000 μM was chosen for subsequent experiments. From Fig. 2c and d, and Supplementary Fig. S9, both individual hydrogen therapy (C_3_N_4_ + AA + US) and hole therapy (C_3_N_4_ + STT + US) displayed distinct antibacterial capability to a certain extent, but hydrogen/hole-combined therapy (C_3_N_4_ + US) demonstrated remarkably higher antibacterial outcomes at the same particle concentration and power density, indicating the hydrogen/hole-combined antibacterial effect.

Considering higher significance of anti-biofilm compared to anti-bacteria in clinic, the sonocatalytic anti-biofilm performance of C_3_N_4_ nanosheets was further evaluated by live/dead and crystal violet staining methods. Similar to the above antibacterial results, live/dead and crystal violet staining results consistently suggested that sonocatalytic therapy with C_3_N_4_ + US had the hydrogen/hole-combined anti-biofilm effect against both *E.coli* and *S.a.* biofilms (Fig. 2e, and Supplementary Figs S10−S13). In order to observe the destruction of biofilm more intuitively, three-dimensional (3D) confocal imaging was used to evaluate the anti-biofilm performance of C_3_N_4_ nanosheets against *E.coli* and *S.a.* biofilms. From Fig. 2e, Supplementary Figs S10 and S11, neither C_3_N_4_ nor US affected the structure of biofilm and biofilm bacterial activity, but hydrogen or hole therapy alone can induce biofilm bacterial death to a certain extent (Fig. 2f and g, Supplementary Figs S12 and S13). However, hydrogen/hole-combined therapy caused remarkably higher anti-biofilm effect as it killed almost all the biofilm bacteria, and also made the biofilm structure become defective. In brief, hydrogen/hole-combined therapy with C_3_N_4_ + US had high efficacies of anti-bacteria and anti-biofilm, killing biofilm bacteria and destroying biofilm structure.

### The mechanism of hydrogen/hole-combined anti-biofilm

**Figure 3.**
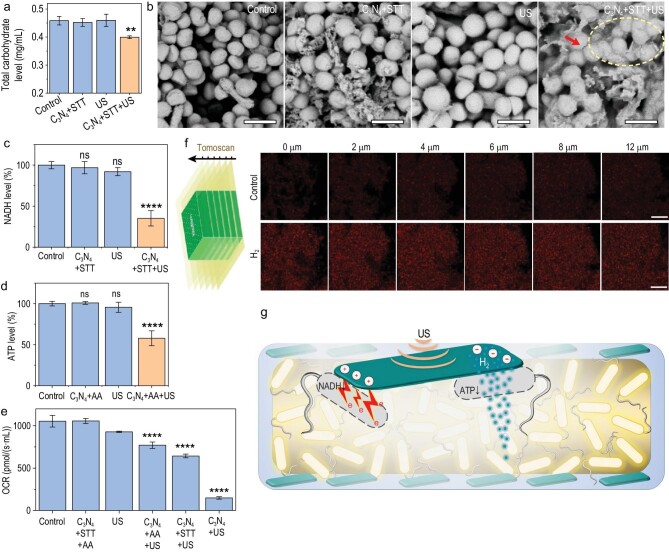
The mechanism of sonocatalytic hydrogen/hole-combined anti-biofilm. Total carbohydrate level in the *S.a.* biofilms with different treatments (*n* = 3 biologically independent samples) (a), SEM images of *S.a.* biofilms (scale bars, 1 μm) (b), NADH level (*n* = 3 biologically independent samples) (c), ATP level (*n* = 3 biologically independent samples) (d) and respiration rates (*n* = 3 biologically independent samples) (e) of biofilm bacteria, schematic diagram of confocal microscope tomoscan and the ratiometric fluorescence with and without addition of saturated hydrogen-rich water (scale bars, 10 μm) (f), and schematic illustration of the sonocatalytic hydrogen/hole-combined anti-biofilm mechanism (g). *P* values were calculated by the one-way ANOVA method (**P < 0.01, ****P < 0.0001; ns, no significant difference).

It is well known that the previously reported photocatalytic/enzymocatalytic antibacterial effect is mainly due to the oxidative damage of catalytically generated ROS to bacterial membrane, which limits the anti-biofilm outcomes [6–12]. The effect and pathway of hydrogen/hole-combined anti-bacteria and anti-biofilm have not been reported. Therefore, the synergistic anti-biofilm mechanism of sonocatalytically generated hydrogen molecules and holes was further investigated here (Fig. 3).

First, STT was used only to sonocatalytically generate holes (C_3_N_4_ + STT + US) to investigate the antibacterial mechanism at the oxidation end. The total carbohydrate and NADH contents within the *E.coli* and *S.a.* biofilms were detected using the corresponding kits. From Fig. 3a and Supplementary Fig. S14, the total carbohydrate content in the C_3_N_4_ + STT + US treated biofilm was significantly less than the other control groups, probably due to the oxidation of polysaccharides within the bacterial wall by sonocatalytically generated holes. Correspondingly, it was clearly visible that bacterial cytoplasm flew out only in the C_3_N_4_ + STT + US group (Fig. 3b, and dashed circle in Supplementary Fig. S15), indicating that the bacterial wall was damaged by holes rather than by C_3_N_4_ and by US [35,36]. Meanwhile, the NADH content in the *S.a.* biofilm bacteria treated with C_3_N_4_ + STT + US was significantly reduced due to the oxidation of holes (Fig. 3c and Supplementary Fig. S16a). As NADH is an important hydrogen/electron carrier in the electron transport chain, a significant decrease of NADH meant the depression of cellular respiration by holes, which was further confirmed by down-regulation of OCR levels in the C_3_N_4_ + STT + US group (Fig. 3e and Supplementary Fig. S16c).

Second, AA was used to sonocatalytically only produce H_2_ to investigate the antibacterial mechanism at the reducing end. Previous researches indicated that hydrogen molecules can regulate the mitochondrial aspiration of damaged and cancerous cells [34,37–41], so we further investigated whether H_2_ can affect the ATP level in biofilm bacteria in this work. Surprisingly, we found that sonocatalytically generated H_2_ (C_3_N_4_ + STT + US) can significantly downregulate the ATP level in the *S.a.* biofilm bacteria (Fig. 3d and Supplementary Fig. S16b), consequently depressing bacterial energy metabolism (Fig. 3e). Taken together, sonocatalytically generated H_2_ and holes jointly inhibited bacterial energy metabolism by the ATP and NADH pathways, respectively (Fig. 3e and Supplementary Fig. S16c). Based on the above pathway analysis, we concluded the mechanism of sonocatalytic hydrogen/hole-combined anti-biofilm, as illustrated in Fig. 3g and Supplementary Fig. S17.

The diffusion of H_2_ is an important factor for destroying the internal structure of biofilm, so we further explored the permeation of H_2_ in bacterial biofilm under confocal microscope imaging taking advantage of our newly-developed ratiometric fluorescent hydrogen nano-probe (NDI-N_3_/Pd@MSN-PEG) [42]. The nano-probe was uniformly dispersed and fixed into *S.a.* bacterial biofilm, and then saturated hydrogen-rich water was added on the biofilm followed by confocal microscope tomoscan imaging. From Fig. 3f, only 2 min after addition of hydrogen-rich water, the ratiometric fluorescence within the whole biofilm was rapidly lightened, indicating that H_2_ can quickly diffuse into the interior of the biofilm. With the increase of time, the fluorescence of the biofilm became stronger and stronger (Supplementary Fig. S18), suggesting increasing amounts of H_2_ penetrated into the biofilm. These results confirmed the hypothesis of hydrogen/hole-combined ‘inside/outside-cooperation’ anti-biofilm (Fig. 3g).

### Infected diabetic wound healing and *in vivo* antibacterial effects

**Figure 4.**
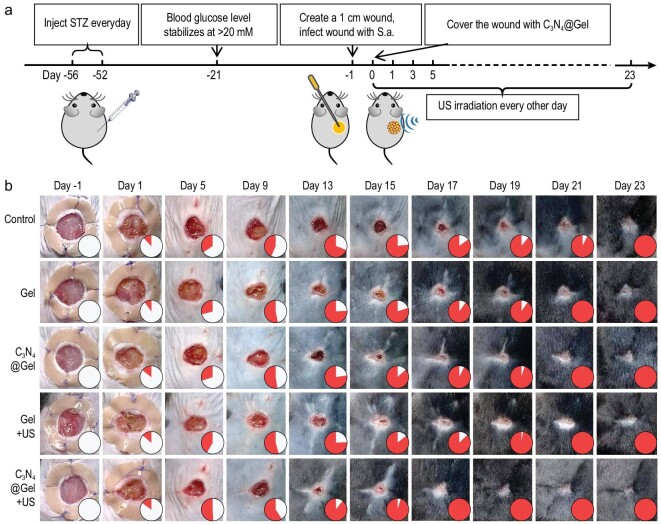
*In vivo* performances of sonocatalytic therapy of infected diabetic wound. The diabetic wound model building and the treatment procedure with C_3_N_4_@Gel + US (a), the digital images of diabetic wounds at different treatment stages (b). Filled color in the inset of figure b corresponds to the percentage of wound healing size. The inside and outside diameters of the circular silicone ring used to fix the skin around the wound were 1 cm and 2 cm, respectively.

Based on the above excellent *in vitro* anti-biofilm outcomes of sonocatalytic therapy, *in vivo* anti-biofilm performance and its effect on infected wound healing were further evaluated on a biofilm infected diabetic wound model. A C_3_N_4_ nanosheets-encapsulated gelatin gel (C_3_N_4_@Gel) was designed for the treatment of the biofilm infected diabetic wound model because the gelatin gel has high biocompatibility [43–45], can fix C_3_N_4_ nanosheets on the surface of the wound and also play a role as US couplant. The diabetic mouse model was first induced by injecting streptozotocin (STZ) every day for five days, and a 1-cm full-thickness excisional wound was established and infected by coating with *S.a.* three weeks after the fasting blood glucose of mice stabilized at >20 mM (Fig. 4a and Supplementary Fig. S19) [46]. After one day, the diabetic wound was coated with the C_3_N_4_@Gel hydrogel and then locally irradiated by US irradiation (1 W/cm^2^, 50% duty ratio, 8 min for twice). From Supplementary Fig. S20, such a dosage of US caused only a slight increase in body and wound temperature which maintained within the safe range (<42°C), minimizing the influence of sonothermal effect on anti-biofilm and wound healing. In addition, C_3_N_4_ nanosheets in a wide concentration range of 0−200 μg/mL did not exhibit obvious cytotoxicity to normal cells, human fibroblasts (HSF) and human immortalized keratin-forming cells (HaCaT), meaning high biocompatibility of C_3_N_4_ nanosheets (Supplementary Fig. S21).

From Fig. 4b and Supplementary Fig. S22, the bacteria infected diabetic wounds took as long as 23 days to heal completely without any treatment. Gel, C_3_N_4_@Gel and Gel + US had a weak healing-promoting effect, possibly due to the fact that the gelatin Gel used can provide a moist environment in favor of wound healing. By comparison, the wounds in the C_3_N_4_@Gel + US group completely healed after treatment for 17 days, reducing the infected diabetic wound healing time by 26%. It indicated that the *in vivo* sonocatalytic hydrogen/hole-combined anti-biofilm based on C_3_N_4_@Gel + US had an important pro-healing effect on the repair of infected diabetic wounds. In addition, the microstructural changes of new skin during the wound healing process were investigated using hematoxylin-eosin (H&E) and Masson's staining methods. As shown in Supplementary Fig. S23, the wounds in the control group always remained larger with obvious inflammatory infiltration and necrotic tissue fragments, and the wounds in the Gel, C_3_N_4_@Gel and Gel + US groups were slightly reduced at the same time points. In contrast, the C_3_N_4_@Gel + US group showed a remarkable decrease in the wound margins, a significant reduction in inflammatory reaction, and a large amount of new granulation tissue under the epidermis, which indicated that sonocatalytic hydrogen/hole-combined anti-biofilm treatment significantly promoted diabetic wound repair.

**Figure 5.**
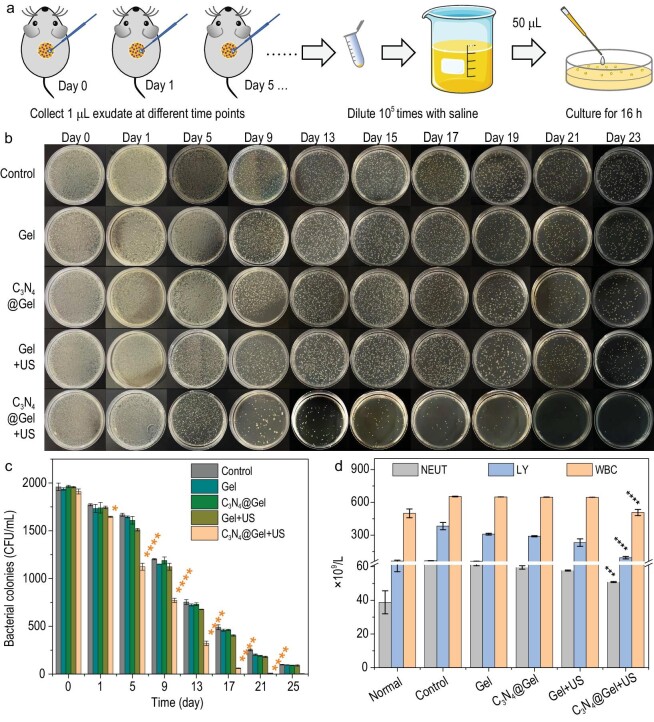
*In vivo* antibacterial performance of sonocatalytic therapy. Schematic diagram of *in vivo* bacterial collection for *in vitro* culture (a), digital pictures of bacterial colonies from diabetic wounds at different time points (b) and corresponding histogram of bacterial colonies (*n* = 3 biologically independent samples) (c), and the contents of blood inflammatory cells after treatment for 23 days (*n* = 3 biologically independent samples) (d). *P* values were calculated by the one-way ANOVA method (*P < 0.1, ***P < 0.001, ****P < 0.0001).

In order to confirm the contribution of anti-bacteria to infected diabetic wound repair, the bacterial amount at the wound was monitored in real time during treatment. As illustrated in Fig. 5a, the exudate at the wound was collected at fixed time points and then diluted 10^5^ times with saline, followed by culture for 16 h on an agar plate. From Fig. 5b and c, diabetic mice cannot effectively eliminate infection by themselves, but sonocatalytic therapy with C_3_N_4_@Gel + US can significantly and gradually reduce the number of bacteria at the wound since day one and received a 97% antibacterial efficacy after treatment for 17 days. At the same time, the systemic inflammation/infection degree was determined by measuring the contents of neutrophils (NEUT), lymphocytes (LY) and white blood cells (WBC) in the blood of mice. From Fig. 5d, sonocatalytic therapy with C_3_N_4_@Gel + US can significantly reduce their contents to normal levels, reflecting outstanding *in vivo* antibacterial outcome.

In addition, in order to verify the biosafety of sonocatalytic therapy with C_3_N_4_@Gel + US *in vivo*, blood samples were collected at the end of treatment for biochemical tests. From Supplementary Figs S24 and S25, all the indicators of the blood samples were maintained within the normal range, suggesting a high biosafety of sonocatalytic therapy. Meanwhile, the mice were humanely euthanized and their main organs including heart, liver, spleen, lung and kidney were extracted and stained by H&E. From Supplementary Fig. S26, all the experimental groups did not cause obvious damage to these major organs, further indicating that the C_3_N_4_@Gel dressing had high biosafety.

## CONCLUSION

In summary, on account of the pathological characteristics and the special microenvironment of the diabetic wound, we developed the C_3_N_4_ nanosheets-encapsulated hydrogel as a sonocatalytic hydrogen/hole production catalyst for synergistic anti-biofilm and promotion of diabetic foot wound healing. Owing to high transmembrane capability, H_2_ was able to penetrate deep into the dense biofilm and efficiently disrupt the biofilm from inside by modulating the bacterial energy metabolism. Meanwhile, the *in situ* generated holes with high oxidative capability facilitated in damaging the surface structure of biofilm and, simultaneously, also affected the electron transport chain. Therefore, the sonocatalytic hydrogen/hole-combined therapy enabled the realization of efficient diabetic wound healing by eradicating biofilm completely from both inside and outside of the biofilm, providing a safe and promising strategy for treatment of deep-seated biofilm and bacteria-infected diabetic foot ulcers.

## METHODS

The details about the synthesis, characterizations and biological performances of C_3_N_4_ nanosheets are in the Supplementary data.

## Supplementary Material

nwad063_Supplemental_Files
