# Supplementary material for: Sonocatalytic hydrogen/hole-combined therapy for anti-biofilm and infected diabetic wound healing
Source: Natl Sci Rev. 2023 Mar 6;10(5):nwad063. doi: 10.1093/nsr/nwad063 (PMC10089581; doi:10.1093/nsr/nwad063)
Supplement: nwad063_Supplemental_Files [file nwad063_supplemental_files.zip › Supplementary data.pdf]

Supplementary Information

Sonocatalytic hydrogen/hole-combined therapy for anti-biofilm and infected diabetic wound healing

Qingqing Xu,[1,2] Shengqiang Chen,[2] Lingdong Jiang,[2] Chao Xia,[2] Lingting Zeng,[2,3] Xiaoqing Cai,[2] Zhaokui Jin,[2] Shucun Qin,[1,*] Wenjiang Ding,[3] and Qianjun He[1,2,3,*]

1. Taishan Institute for Hydrogen Biomedical Research, School of Basic Medical Sciences, The Second Affiliated Hospital of Shandong First Medical University & Shandong Academy of Medical Sciences, Tai'an 271000, China

2. Guangdong Key Laboratory for Biomedical Measurements and Ultrasound Imaging, School of Biomedical Engineering, Medical School, Shenzhen University, Shenzhen 518060, China

3. Shanghai Key Laboratory of Hydrogen Science & Center of Hydrogen Science, School of Materials Science and Engineering, Shanghai Jiao Tong University, Shanghai 200240, China

* Corresponding Author. Qianjun He (nanoflower@126.com), Shucun Qin (shucunqin@hotmail.com)

METHODS

Synthesis of C_3N_4 nanosheets

10 g of urea was dispersed in 5 mL of deionized water, and then added into an alumina crucible, which was placed in a muffle furnace and then heated to 550 °C at a rate of 0.5 °C/min and maintained for 3 h. After reaction, the resulting solid powder was cooled to room temperature and washed with deionized water and anhydrous ethanol in turn, and the precipitate was re-dispersed in deionized water. Then the suspension (2 mg/mL) was treated with an ultrasonic crusher for 2 h (150 W, 80% duty cycle) and then C_3N_4 nanosheets were collected by centrifugation and washed with deionized water for two times. The yield of C_3N_4 nanosheets achieved at 28.4%.

Characterization of C_3N_4 nanosheets

The morphology and size of C_3N_4 nanosheets were measured by SEM (Thermo APREO-S), STEM (APREO, FEI), and AFM (MFD-3D, Oxford). The crystal phase was characterized by XRD (M21X, Cu Kα radiation, λ=0.154178 nm) with the accelerating voltage and current of 40 kV and 200 mA, respectively. The piezoelectric effect of C_3N_4 nanosheets was characterized by using Piezo-response force microscopic (PFM). UV spectrum of C_3N_4 nanosheets was recorded on a UV spectrophotometer (Genesys 10S). The Mott-Schottky plot of C_3N_4 nanosheets was determined on a CHI 660D electrochemical station (Shanghai Chenhua, China).

In vitro cytotoxicity assessment

Human fibroblasts (HSF) and human immortalized keratin-forming cells (HaCaT) were inoculated in 96-well plates at a density of 1×10^4 cells per well, and then were

co-incubated with different concentrations of C_3N_4 nanosheets (0–200 μg/mL) for 12 h. Finally, the cytotoxicity of C_3N_4 nanosheets to the cells was detected using the CCK-8 colorimetric assay.

Bacteria and biofilms culture

A single colony was found out in the three-zone plate covered with *E.coli*/*S.a.*, and then two colonies were selected and transferred into 10 mL LB medium (Sangon Biotech) for 6 h incubation in shaker (37 °C, 200 rpm). The optical density (OD) of bacterial solution at 600 nm was measured on a microplate reader (Bio-Tek ELx800), and the culture was stopped until OD=0.2. Finally, the bacterial solution was centrifuged (5000 rpm for 5 min) and washed twice with PBS and then re-suspended in saline for use. As for biofilm culture, *E.coli*/*S.a.* bacterial suspension (2×10^8 CFU/mL) was ad ded to the LB medium (3 mL) and then incubated at 37 °C for 72 h. The culture medium was refreshed once every 36 h.

Detection of sonocatalytic hydrogen production and anti-bacteria *in vitro*

C_3N_4 nanosheets (8 mg) were dispersed in the suspension of *E.coli* or *S.a.* (2×10^8 CFU/mL, 4 mL) and then the bacterial suspension containing C_3N_4 nanosheets was stimulated (1.0 MHz, 1 W/cm^2, 50% duty cycle) by a medical ultrasound physiotherapy instrument (2773AS, Chattanooga Co.). Hydrogen production was monitored by gas chromatography (Agilent 7890B, USA) every 2 min. At the same time, the bacterial suspensions were diluted 1×10^5 times with 0.9% saline, and 50 μL of the diluted bacterial culture was spread onto an agar plate for incubation in shaker for 16 h at 37 °C. Finally, the bacterial colony number in each agar plate was counted. The same antibacterial activity evaluation procedure was used in the *in vitro* antibacterial experiments involving sacrificial agents (AA and STT).

In vitro antibacterial biofilm assay

Similar to the above antibacterial assay, C_3N_4 nanosheets and sacrificial agents (AA or STT) were added to the *E.coli* or *S.a.* bacterial biofilm followed by US irradiation. After incubation at 37 °C for 16 h without shaking, the biofilms were washed two times with PBS to remove floating bacteria and materials, and then biofilm bacterial activity was evaluated by crystal violet staining and confocal microscopy imaging methods.

As for crystal violet staining method, the biofilm was immediately fixed at 4 °C for 15 min by addition of 200 μL methanol, followed by natural dry at room temperature. Next, 200 μL of 1% crystalline violet solution (Macklin, AR) was added to each well, stained for 15 min at room temperature and then aspirated out. The biofilm was rinsed with PBS to remove excessive dye and dried again upside down on paper at 37 °C. After complete drying, 200 μL of glacial acetic acid solution (Macklin, AR, 33%) was added to each well and incubated at 37 °C for 30 min. Finally, the OD_{590nm} was measured on the microplate reader and the color of biofilm in each well was recorded using digital camera.

As for confocal microscopy imaging, the biofilm was washed twice with PBS to remove floating bacteria and materials, then 3 μL of fluorescent dye (Syto-9:PI=1:1) was added and incubated with the biofilm at 37 °C for 20 min in the dark. Finally, fluorescent images of bacterial biofilms were recorded using confocal laser scanning microscopy (ZEISS LSM880).

Antibacterial mechanism assay

E.coli and *S.a.* bacteria were first cultured into corresponding biofilms in six-well plates, then washed twice with PBS and treated with C_3N_4 nanosheets and sacrificial agents (AA or STT) followed by US irradiation. Finally, the biofilm was placed in a respirometer test well to detect bacterial oxygen consumption rate (Oroboros Instruments,

O_2k-Respirometer). On the other hand, the biofilm was lysed and the supernatant was collected to detect the concentrations of ATP, carbohydrate and NADH using an ATP kit (Beyotime Biotech), a total carbohydrate assay kit (Sigma-Aldrich) and a NADH/NAD^+ kit (Solarbio, BC2715), respectively.

Hydrogen permeation biofilm experiments

The fluorescent ratiometric hydrogen nanoprobe (50 μg/mL) was incubated with *S.a.* bacteria for 72 h to form the nanoprobe-embedding bacterial biofilm in a confocal glass dish. After washing off the floating bacteria and free nanoprobe with PBS, 2 mL of saturated hydrogen-rich water was added into the dish, and confocal laser scanning microscopy imaging was performed at fixed time points.

Bacteria-infected diabetic wound model establishment

All the animal experiments followed the protocols approved by the Animal Care and Use Committee of Shenzhen University. Male C57BL/6J mice (6-7 weeks old, 25–30 g) were purchased from Guangdong Medical Laboratory Animal Center of Experimental Animal. Mice were maintained at 22–25 °C with a 12 h light/dark cycle and provided with normal food and drinking water. After normal feed for three days, a high-fat diet was served for two weeks to induce insulin resistance. Then, streptozotocin (55 mg/kg/day, Sigma-Aldrich) was injected intraperitoneally (i.p.) into mice for five consecutive days. The blood glucose level of mice was monitored every seven days.

Diabetic wound establishment was initiated after the fasting blood glucose concentration of mice reached >20 mM and then remained stable for three weeks. A full-thickness excisional wound was made on the dorsal surface of each mouse by using a 1 cm biopsy punch. The skin around the wound was fixed with a circular silicone ring to avoid the influence of natural contraction of skin on wound healing. Finally, diabetic wound

was infected by dropping 30 μL of *S.a.* suspension (1.0×10^{10} CFU/mL) on the freshly created wound.

In vivo wound-healing and antibacterial evaluation

Firstly, a gelatin gel was prepared by dissolving gelatin powder (2 g) into water (8 mL) at 60 ˚C, and then mixed with C_3N_4 nanosheets to obtain the fresh C_3N_4@Gel, which was immediately coated on the *S.a.* infected diabetic wound after 1-day bacterial infection followed by US irradiation twice (8 min every time, 1 W/cm^2, 50% duty cycle) every other day. At fixed time points, digital photos of wounds were taken and the wound size was calculated using ImageJ. On day 9, 19 and 23, one mouse in each group was mercifully executed, and the skin tissue surrounding the whole wound was carefully collected and fixed with 10% formalin to prepare the pathological slides. The tissue sections were stained by H&E and Masson's trichrome, and their histological images were acquired on an optical microscope (Nikon-2000U, Japan).

On day 0, 1, 5, 9 and 13, 1 μL of exudate at the wound was collected with a 1 μL inoculation ring, and on day 15, 17, 19, 21 and 23, 1 μL sterile saline was dropped at the wound to collect bacteria using the 1 μL inoculation ring. Then, the exudate was diluted 1×10^5 times and 50 μL of the diluted bacterial culture was spread onto an agar plate and incubated at 37 °C for 16 h. Finally, the bacterial colonies in each agar plate were imaged and counted to compare the *in vivo* antibacterial performances of different treatments.

In vivo sonothermal effect assay of C_3N_4@Gel

The freshly prepared C_3N_4@Gel was coated on the surface of wound and then irradiated by US for 9 min on the ultrasonic physiotherapy apparatus (1.0 MHz, 1 W/cm^2, 50% duty cycle). At intervals of 1 min, thermal images of mice were captured using a NIR thermal imaging camera (Fluke, Ti400), and temperature data at the wound, in the body

and in the environment were recorded as well.

In vivo assessment of liver/kidney functions and systemic toxicity

On the last day of treatment, major organs of mice including the heart, liver, spleen, lung, and kidney were collected, sliced, and stained with H&E to evaluate the *in vivo* safety of C_3N_4 nanosheets and US. At the same time, blood was collected and assessed using a biochemical analyzer (iMagic-M7) and a blood cell analyzer (BC-31s, Mindray).

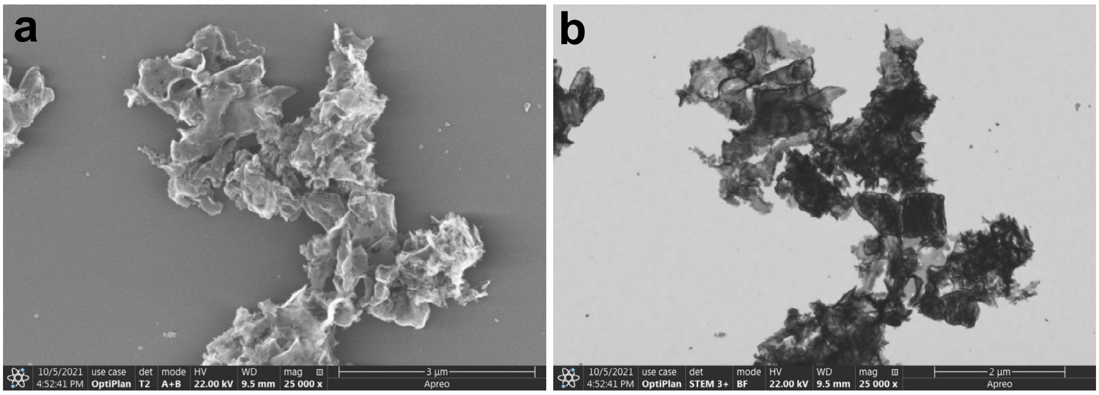

Figure S1. The SEM (**a**) and corresponding STEM (**b**) images of bulk C_3N_4.

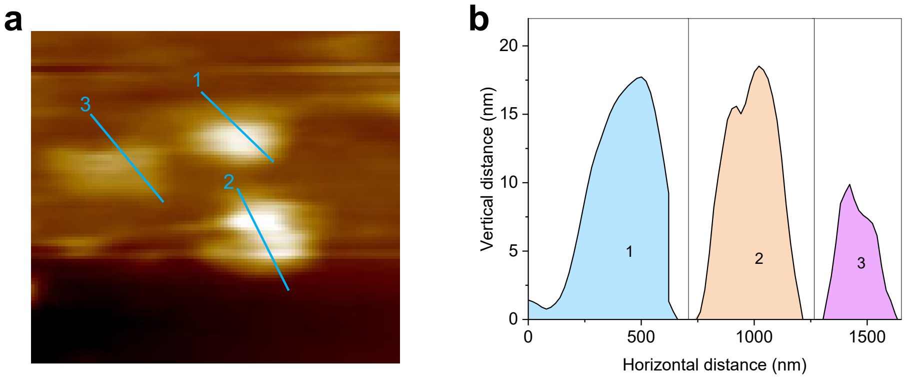

Figure S2. The AFM (**a**) images and dimensional distribution (**b**) of C_3N_4.

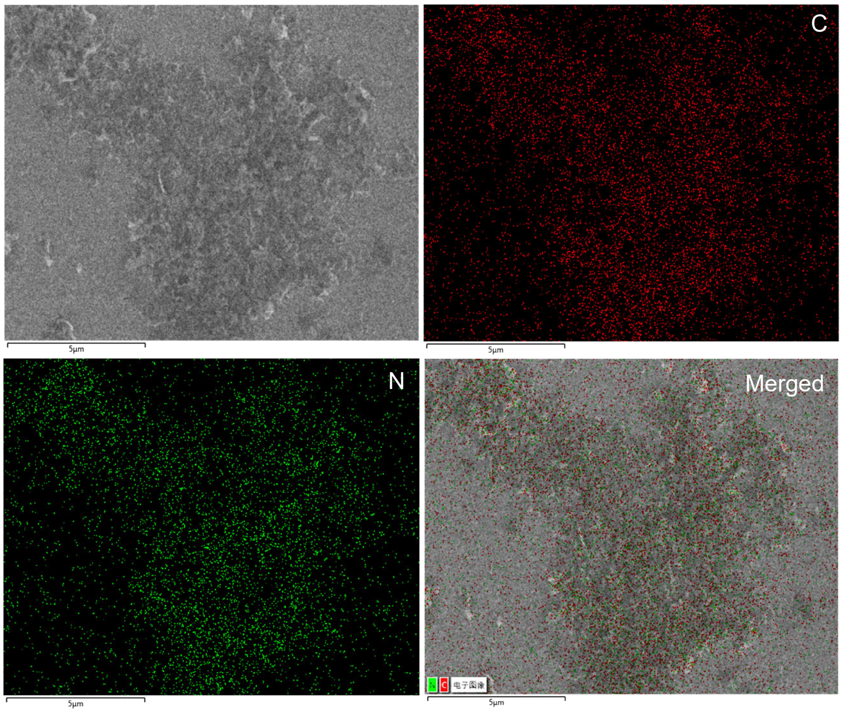

Figure S3. The STEM image and elemental distribution mapping of C_3N_4 nanosheets.

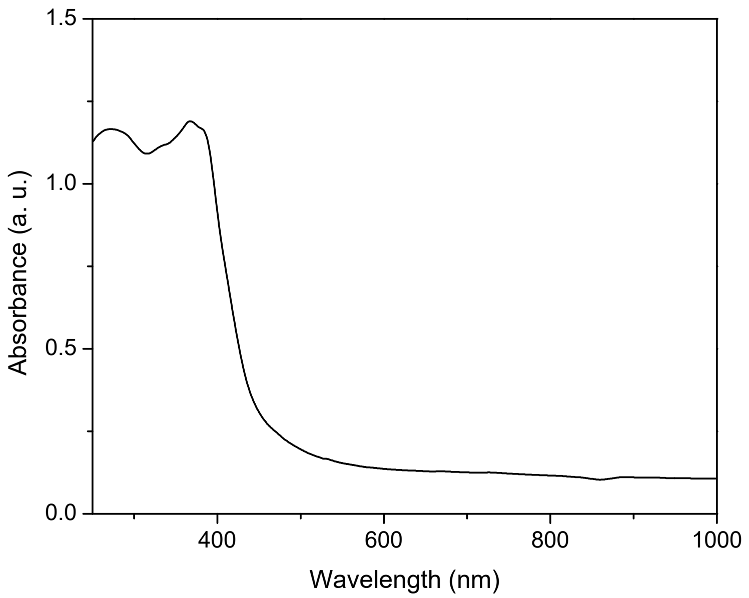

Figure S4. The UV absorption spectrum of C_3N_4 nanosheets.

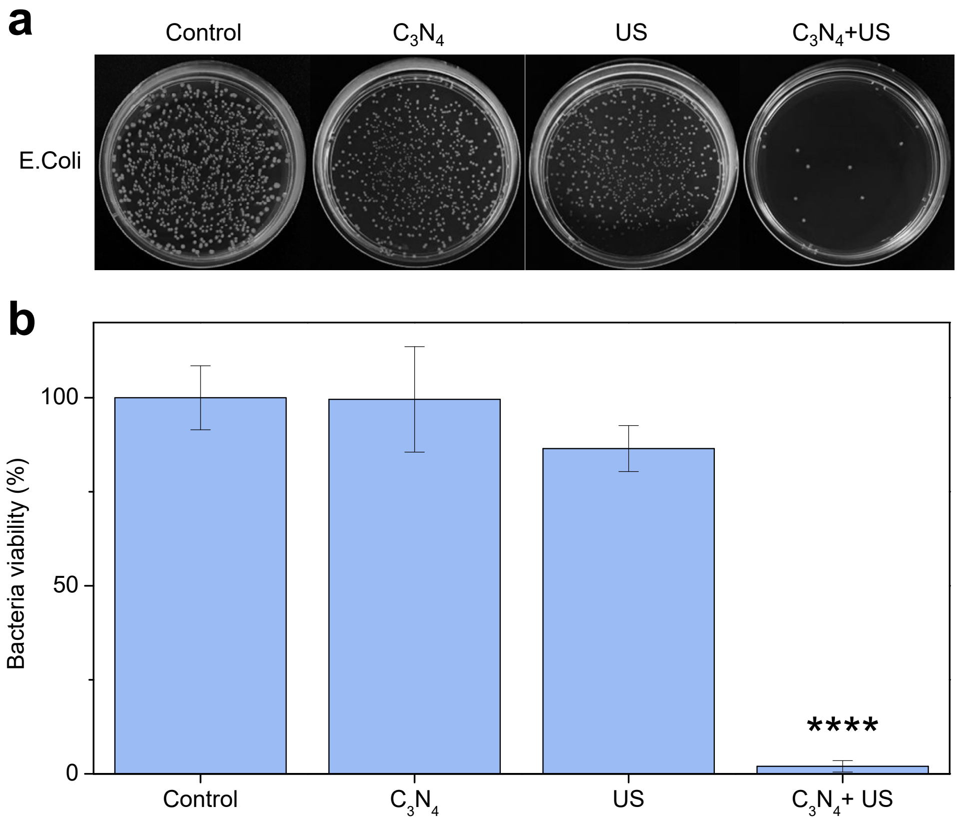

Figure S5. Digital photographs (**a**) and corresponding statistical analysis (**b**) of the hydrogen/hole-combined antibacterial performance of C_3N_4 against *E.coli* (*n*=3 biologically independent samples). *P* value was calculated by the one-way ANOVA method (**** *P*<0.0001).

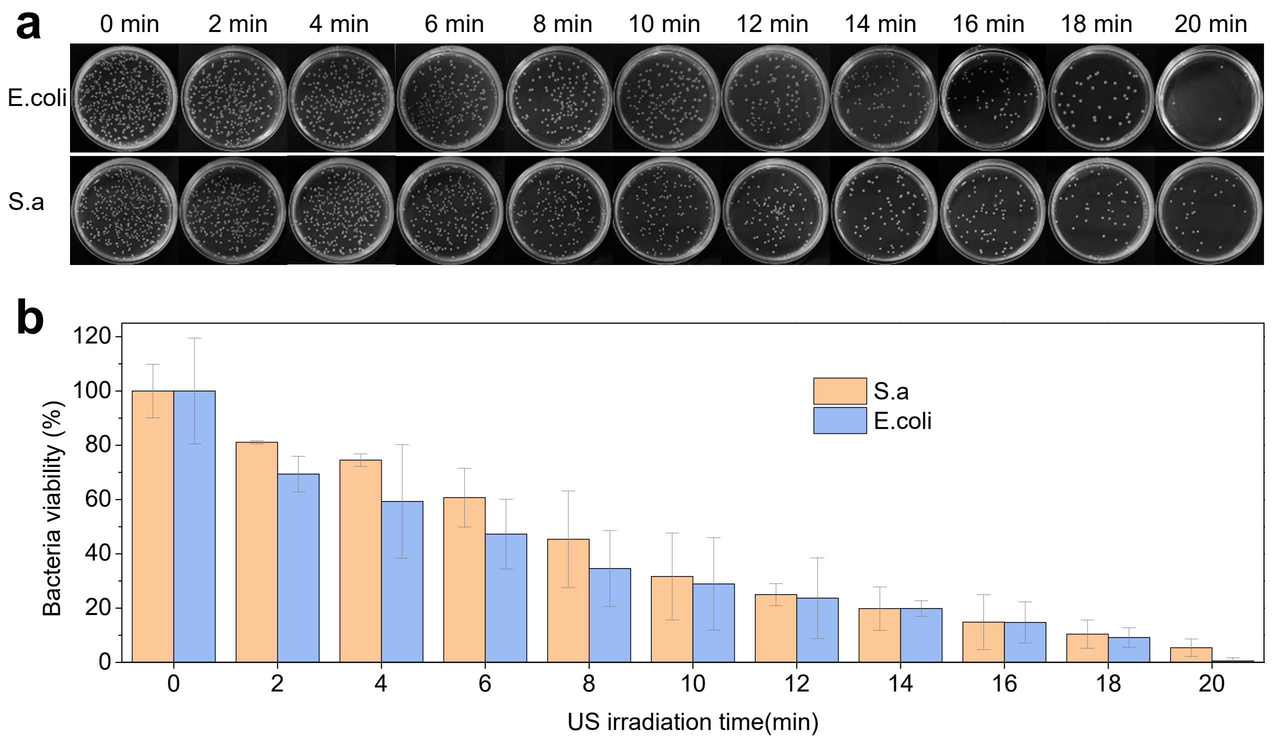

Figure S6. Antibacterial digital photos (**a**) and corresponding quantitative analysis (**b**) over 20 min at the US power density of 1 W/cm^2 with 50% duty ratio (*n*=3 biologically independent samples).

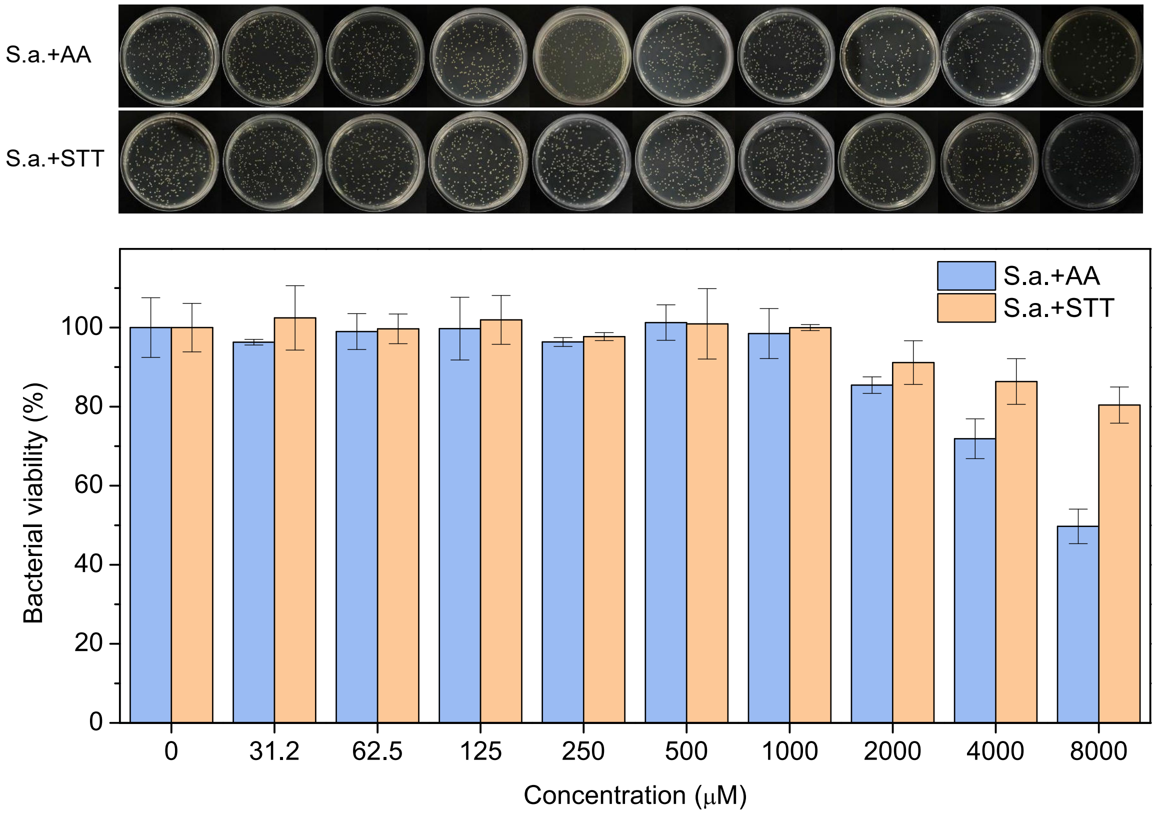

Figure S7. Cytotoxicity of AA and STT against *S.a.* bacteria (*n*=3 biologically independent samples).

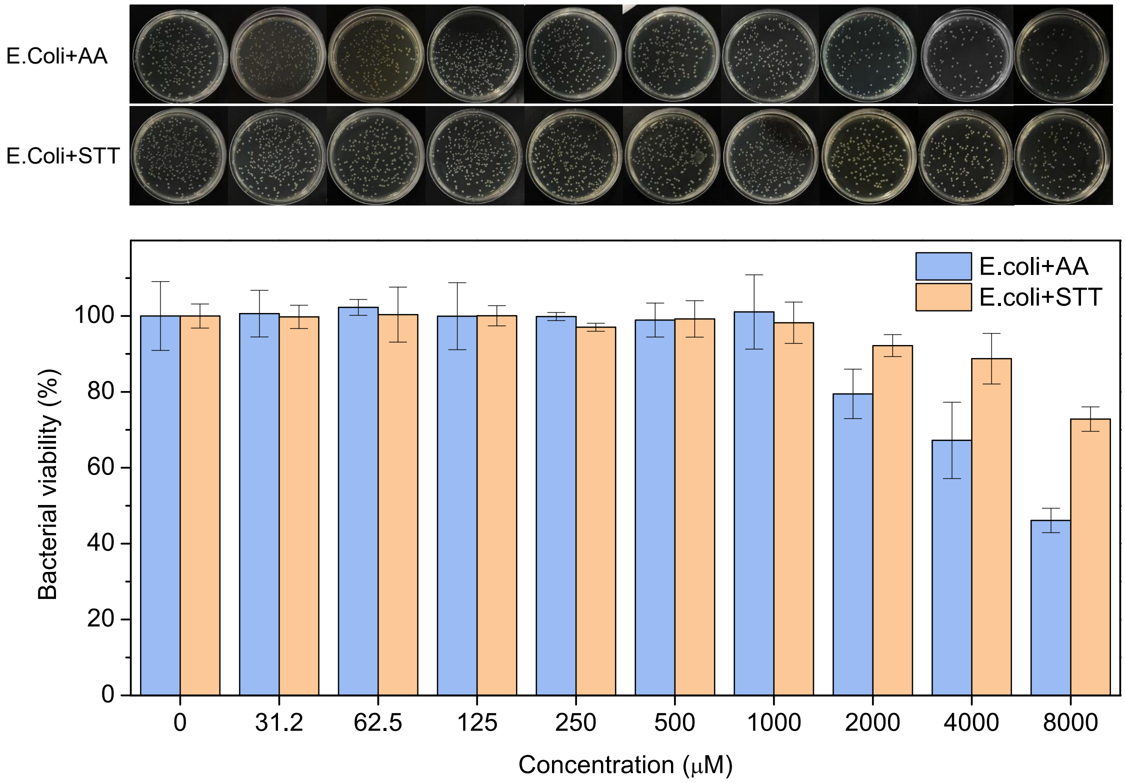

Figure S8. Cytotoxicity of AA and STT against *E.coli* bacteria (*n*=3 biologically independent samples).

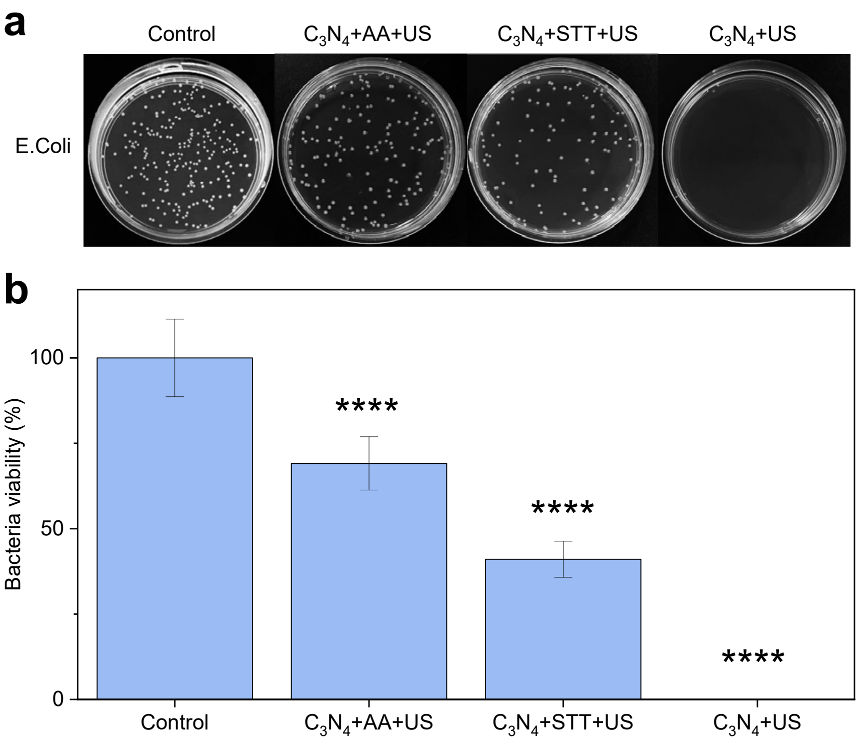

Figure S9. Digital photographs (**a**) and corresponding statistical analysis (**b**) of the contributions of individual hydrogen and hole therapy of *E.coli* (*n*=3 biologically independent samples). *P* values were calculated by the one-way ANOVA method (**** $P<0.0001$).

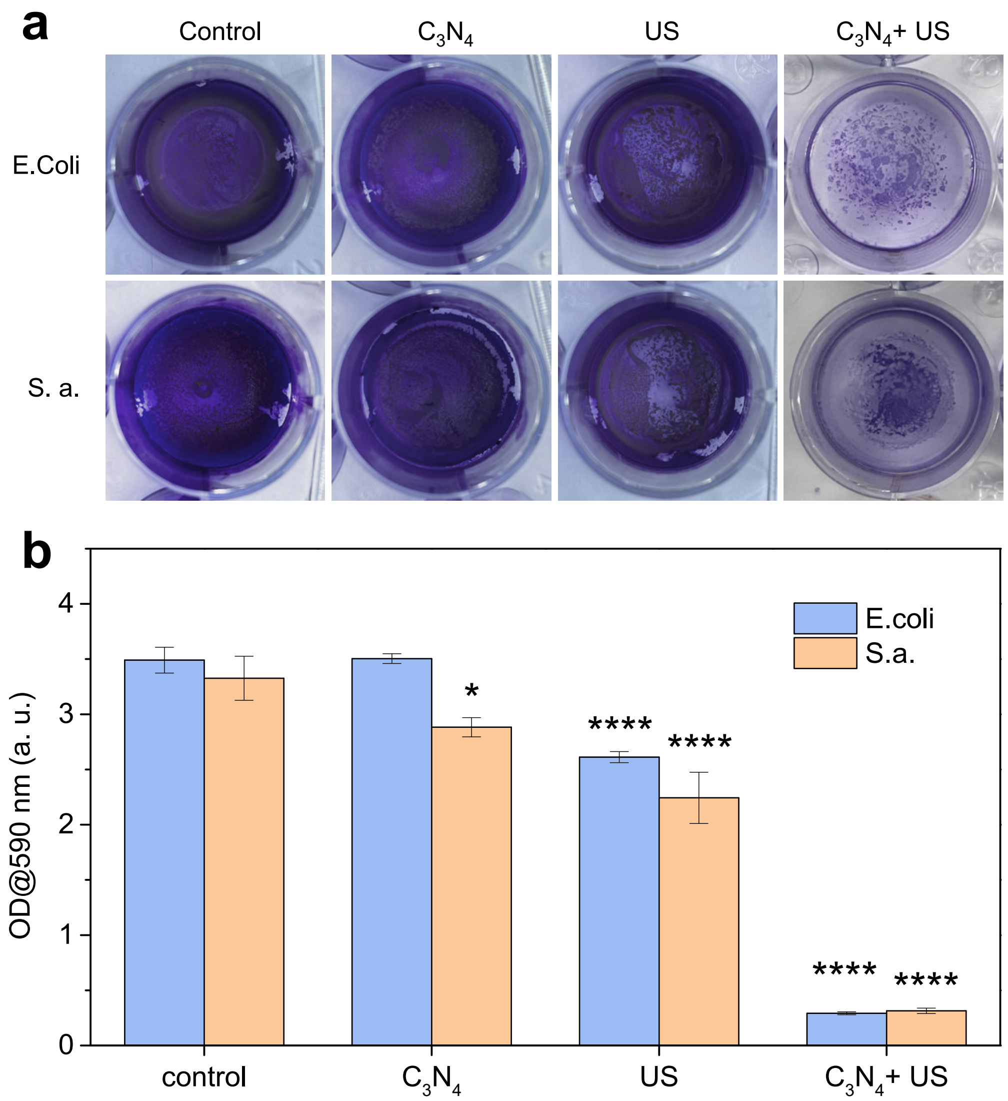

Figure S10. Digital photographs (**a**) and corresponding statistical analysis (*n*=3 biologically independent samples) (**b**) of the hydrogen/hole-combined anti-biofilm performance of C_3N_4 nanosheets against *E.coli* and *S.a.* biofilms. *P* values were calculated by the one-way ANOVA method (* *P*<0.1, **** *P*<0.0001).

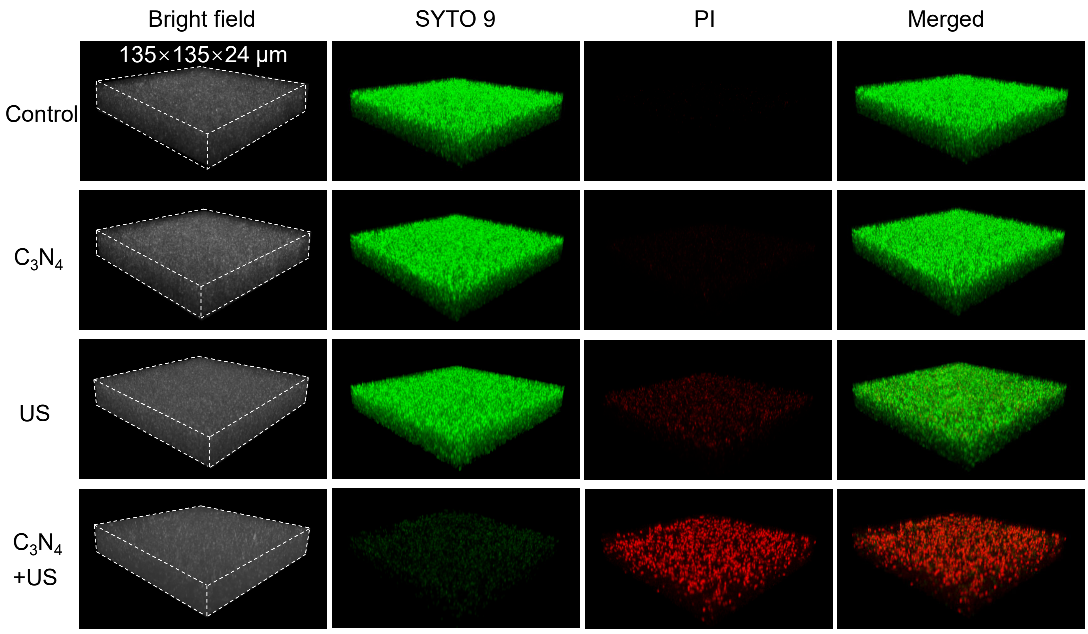

Figure S11. The anti-biofilm activity against *E.coli* assessed by 3D confocal imaging.

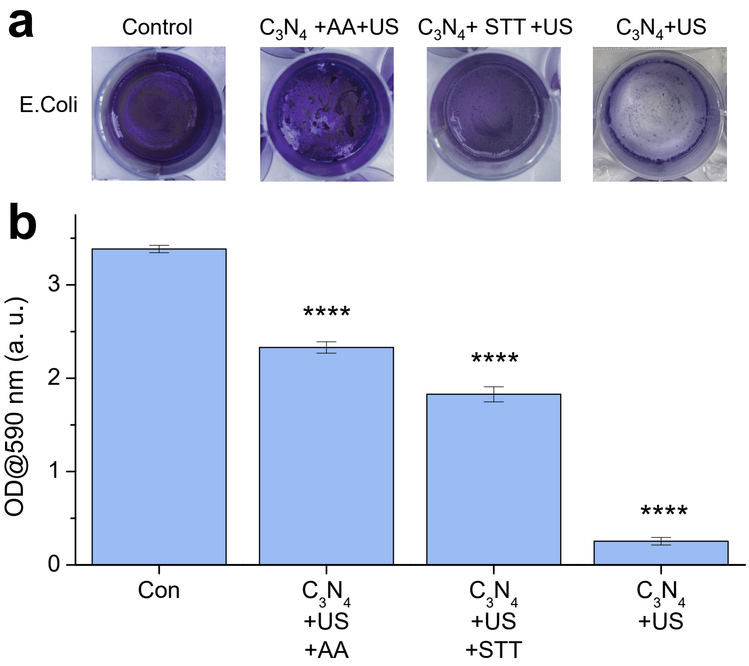

Figure S12. Digital photographs (**a**) and corresponding statistical analysis (*n*=3, biologically independent samples) (**b**) of the hydrogen/hole-combined anti-biofilm performance of C_3N_4 nanosheets against *E.coli*. Con, control. *P* values were calculated by the one-way ANOVA method (**** P<0.0001).

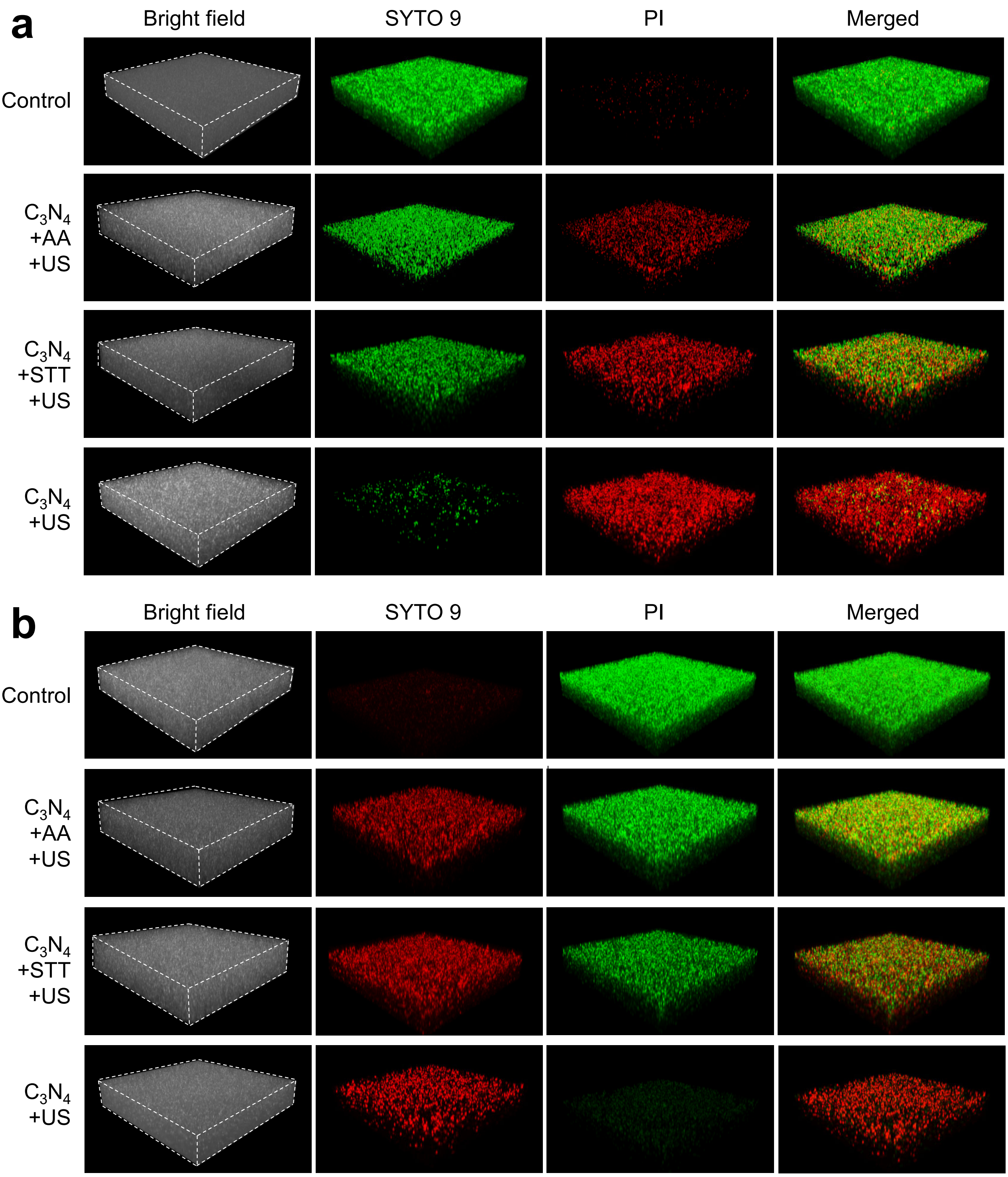

Figure S13. The anti-biofilm contributions of individual hydrogen and hole therapy against *S.a.* (**a**) and *E.coli* (**b**) assessed by 3D confocal imaging.

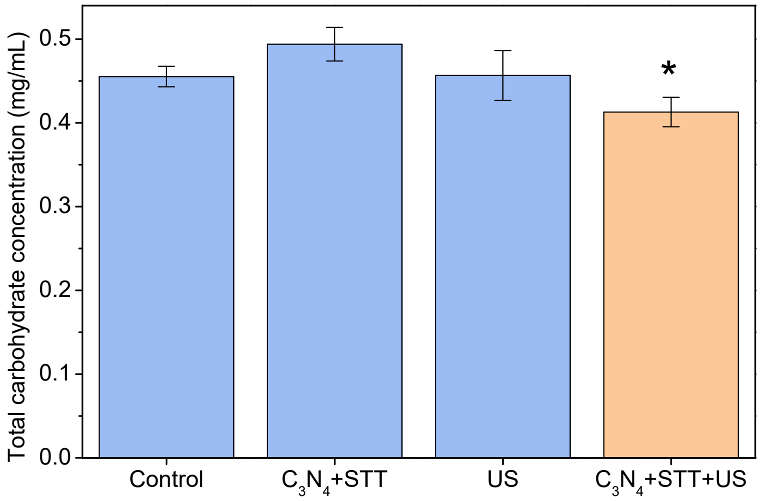

Figure S14. Total carbohydrate level (*n*=3, biologically independent samples) in *E.coli* biofilms with different treatments. *P* value was calculated by the one-way ANOVA method (*P<0.1).

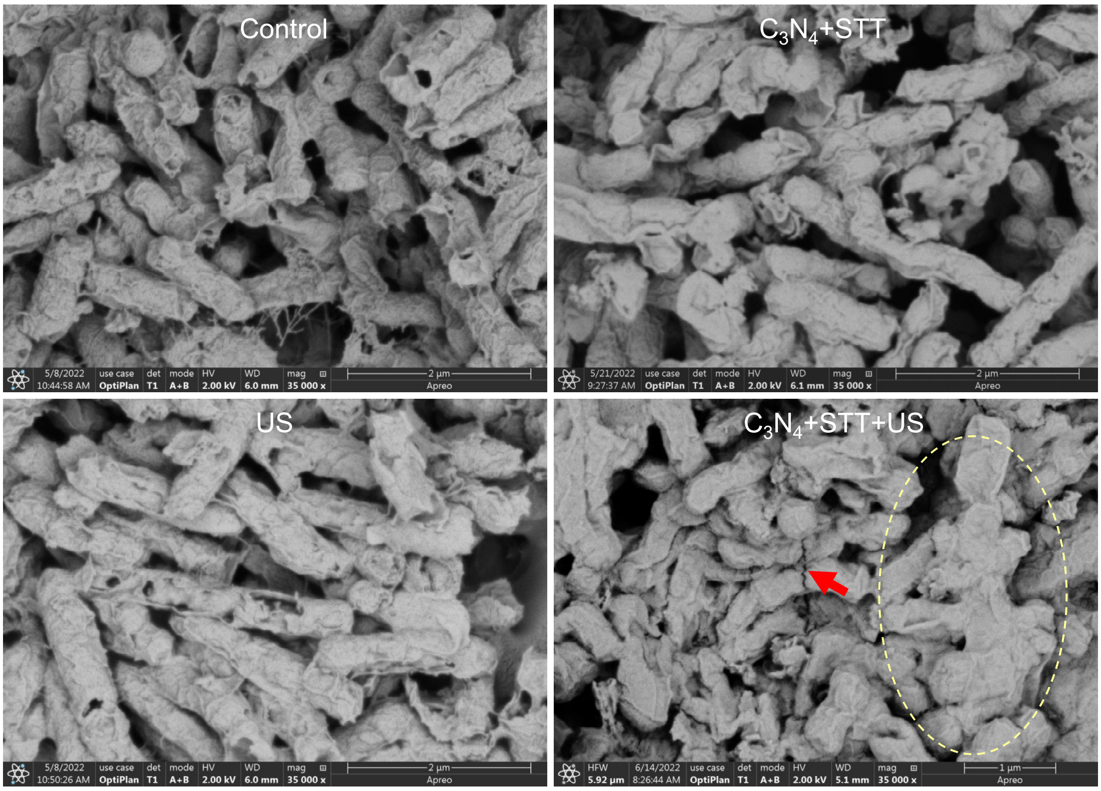

Figure S15. SEM images of *E.coli* biofilms in different treatment groups. The bacteria in the C_3N_4+STT+US group deformed and fractured (as indicated by red arrow), and cytoplasm flew out and covered on the surface of bacterial fragments (as indicated by dashed circle).

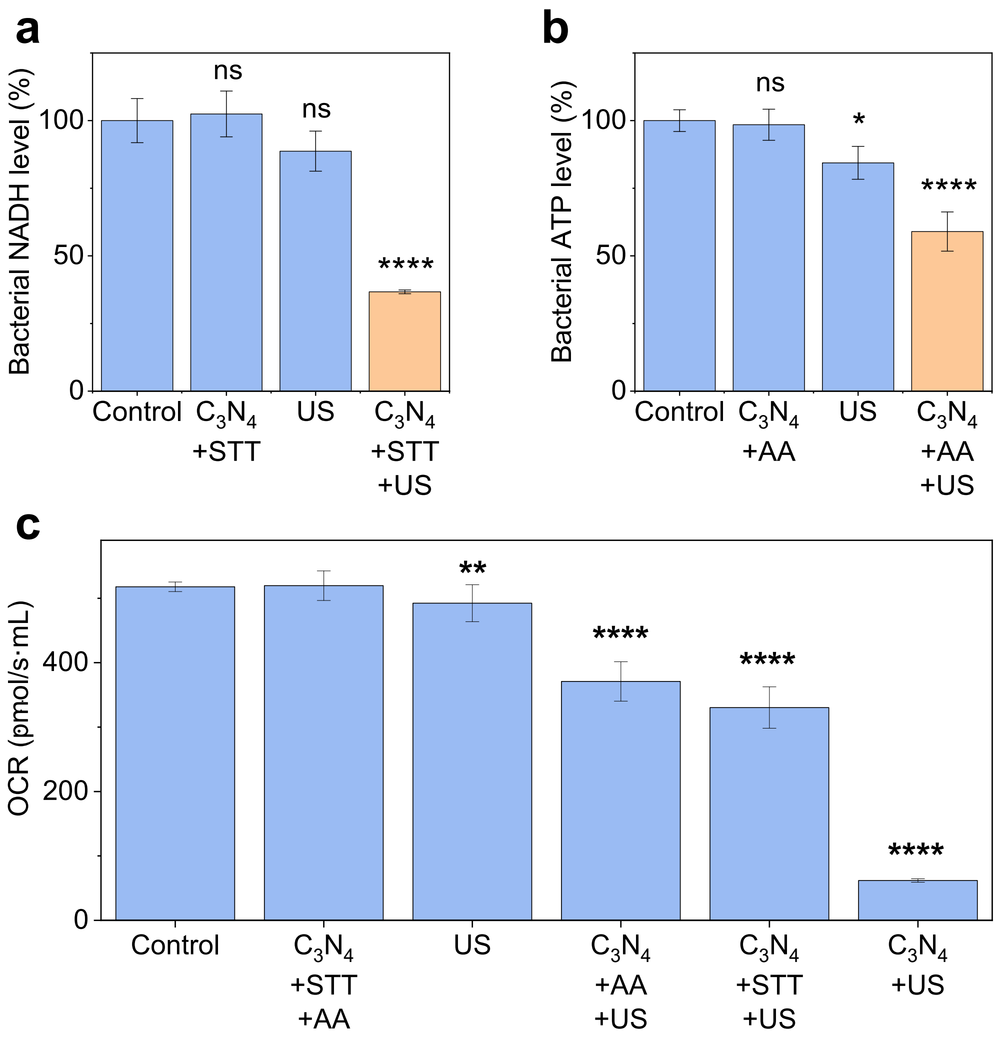

Figure S16. NADH level (**a**) and ATP level (**b**) and respiration rates (**c**) of bacteria within *S.a.* biofilms in different treatment groups. *n*=3, biologically independent samples. *P* values were calculated by the one-way ANOVA method. (**P*<0.1, ***P*<0.01, *****P*<0.0001).

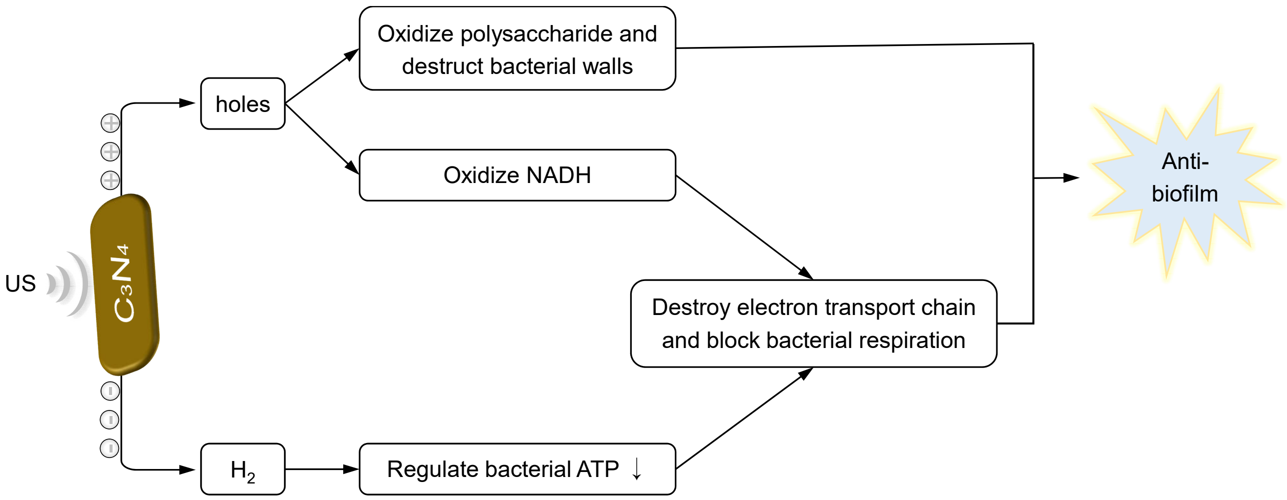

Figure S17. The pathway of sonocatalytic hydrogen/hole-combined anti-biofilm.

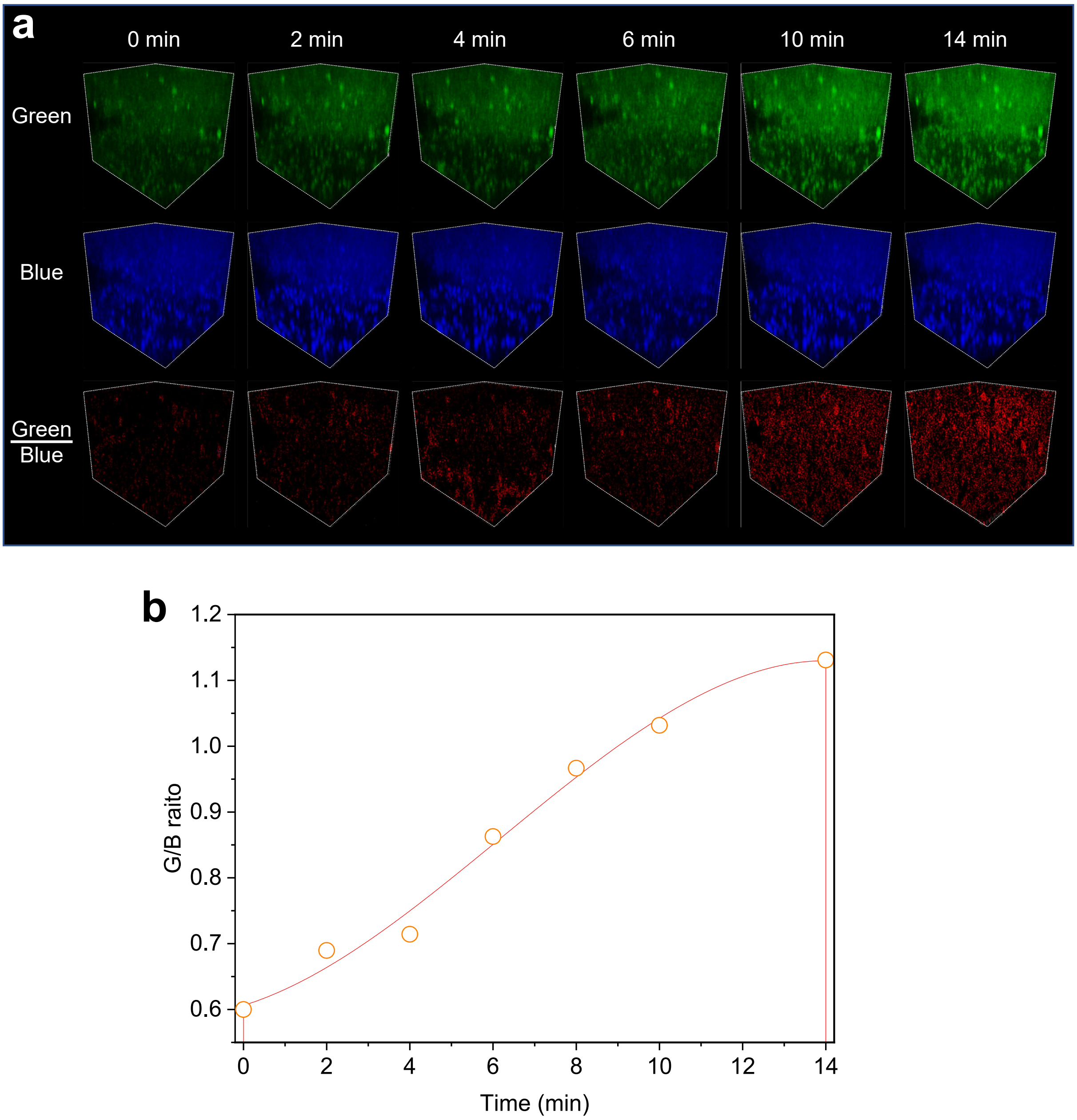

Figure S18. The fluorescence ratio (G/B) change inside the fluorescent nanoprobe-embedded *S.a.* biofiom within 14 min after addition of hydrogen-rich water.

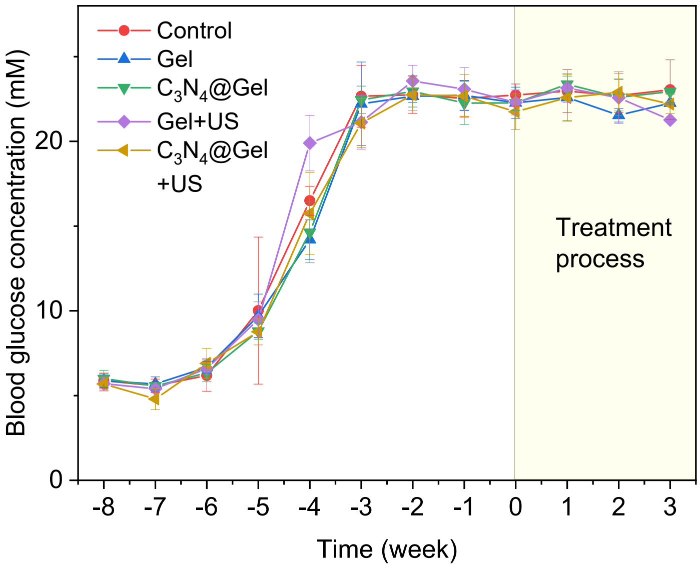

Figure S19. Fasting blood glucose changes in mice before and after treatment (*n*=5, biologically independent samples).

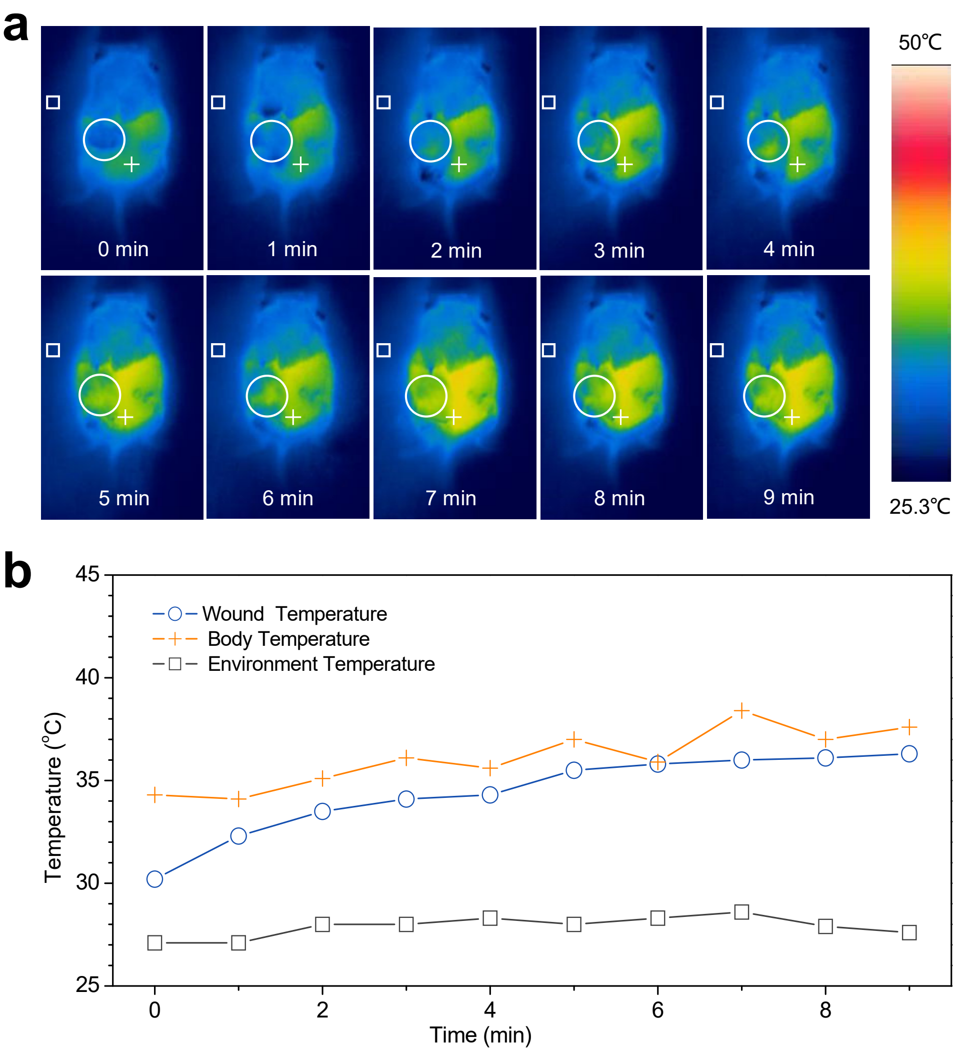

Figure S20. US-induced temperature increase at the wound (O), in the body (+) and in the environment outside the body (□) (**a**), and corresponding quantitative analysis (**b**).

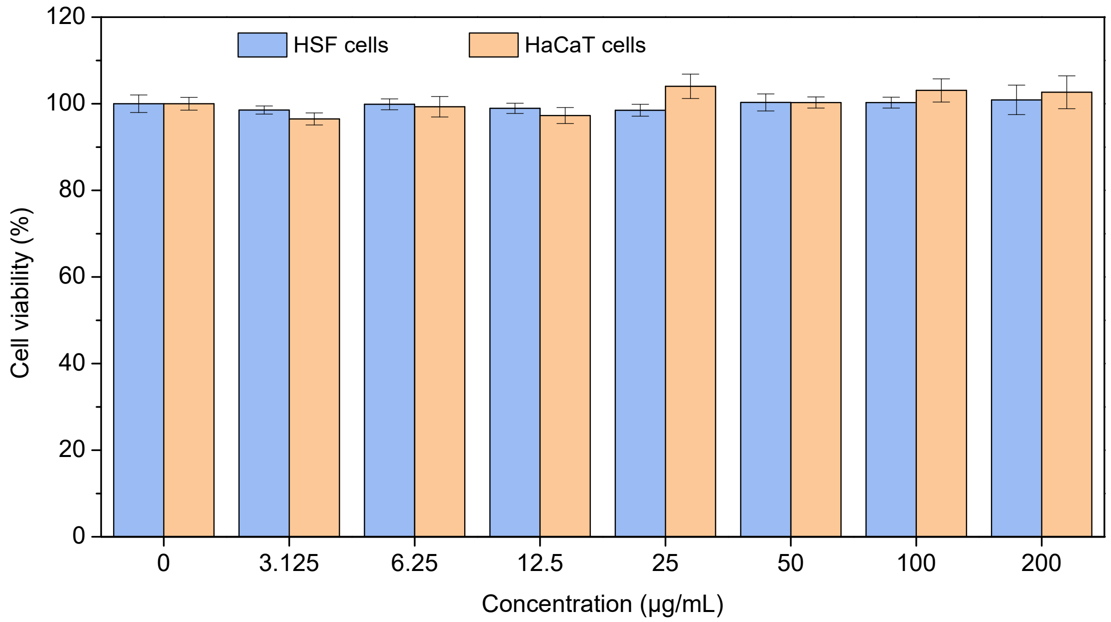

Figure S21. Cytotoxicity of C_3N_4 nanosheets to HSF and HaCaT cells (*n*=6, biologically independent samples).

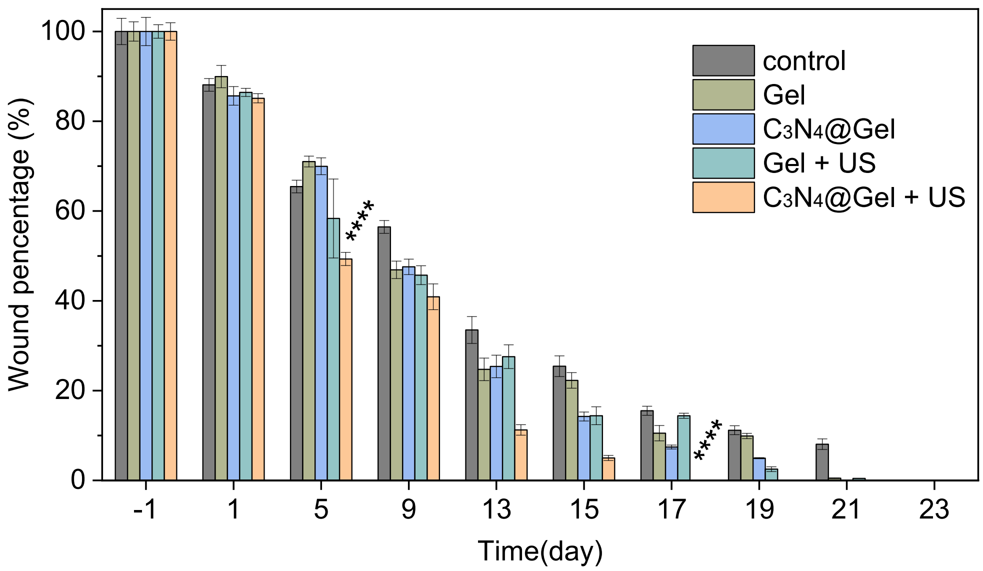

Figure S22. Statistical analysis of wound percentage in mice after treatment for different time durations (*n*=5, biologically independent samples). *P* values were calculated by the one-way ANOVA method (*****P*<0.0001).

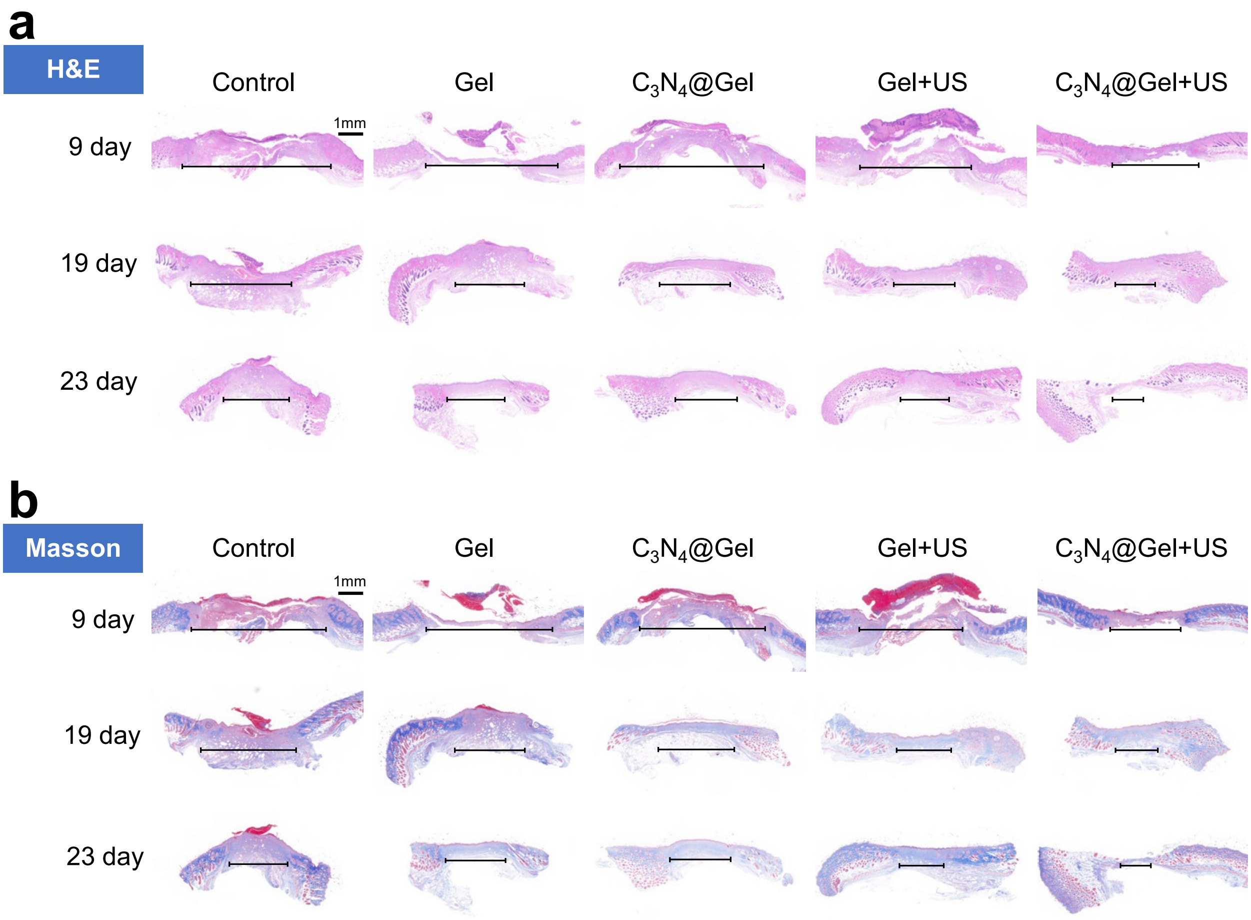

Figure S23. Histological assessment of wounds on day 9, 19 and 23 by H&E (**a**) and Masson's (**b**) stained images.

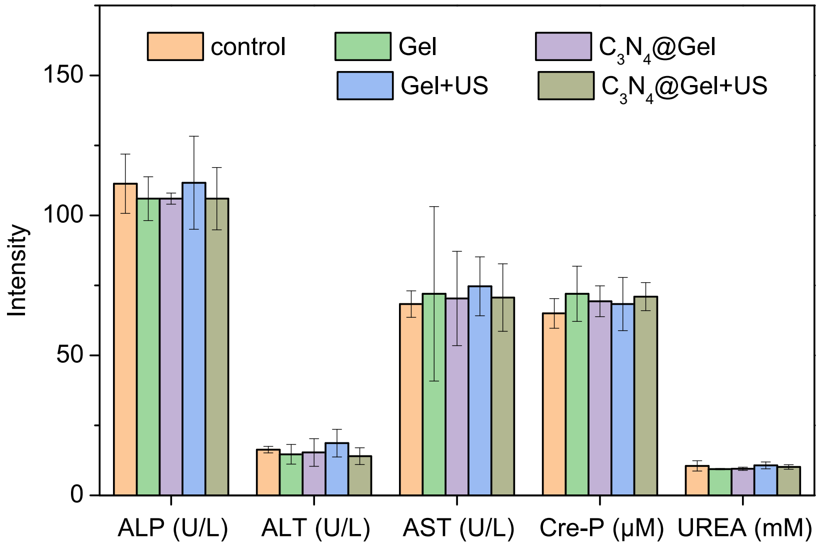

Figure S24. Blood biochemical analysis of liver/kidney functions in mice. ALP: alkaline phosphatase; ALT: alanine aminotransferase; AST: aspartate aminotransferase; Cre-P: creatinine; UREA: urea nitrogen (*n*=7, biologically independent samples).

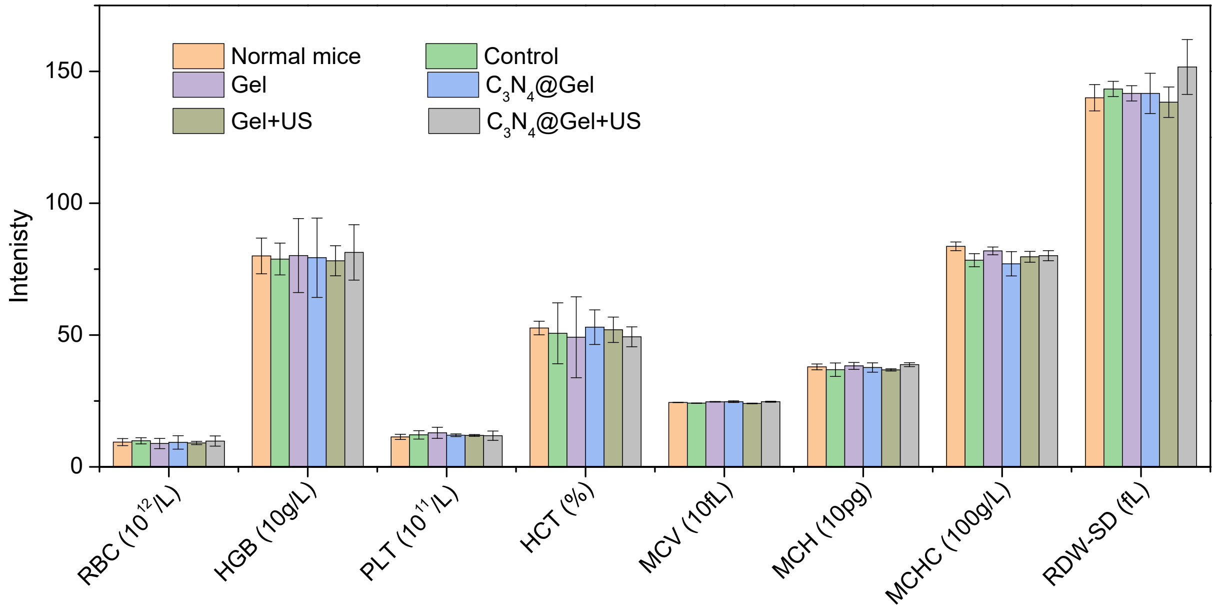

Figure S25. Routine blood biochemical parameters of mice. RBC: red blood cell content; HGB: hemoglobin concentration; PLT: platelet content; HCT: hematocrit; MCV: mean red blood cell volume; MCH: mean corpuscular hemoglobin; MCHC: mean corpuscular hemoglobin concentration; RDW-SD: standard deviation of red blood cell distribution width (n=3, biologically independent samples).

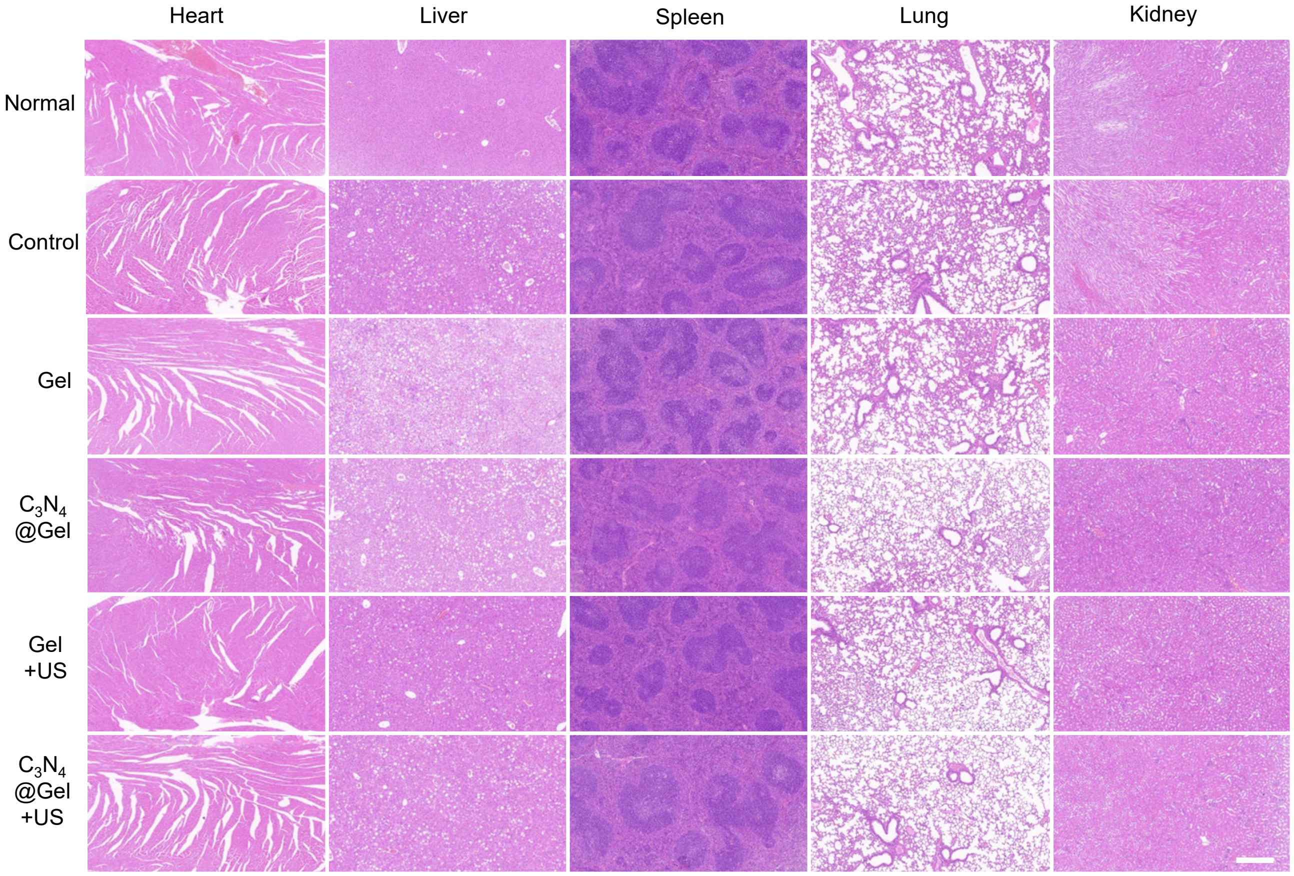

Figure S26. Histological examination of major organs (heart, liver, spleen, lung and kidney) by H&E staining method after different treatments. Scale bars represent 500 μm.
