# Supplementary material for: Sonocatalytic hydrogen/hole-combined therapy for anti-biofilm and infected diabetic wound healing
Source: Natl Sci Rev. 2023 Mar 6;10(5):nwad063. doi: 10.1093/nsr/nwad063 (PMC10089581; doi:10.1093/nsr/nwad063)
Supplement: nwad063_Supplemental_Files [file nwad063_supplemental_files.zip › Teaser text.docx]

Teaser text

A concept of sonocatalytic hydrogen/hole-combined ‘inside/outside-cooperation’ anti-biofilm is proposed for efficient hydrogen generation and local polysaccharide/NADH oxidation, which inhibits bacterial aspiration inside biofilm and destroys the surface structure of biofilm, respectively.
